# Subcutaneous methotrexate compared with oral methotrexate in rheumatoid arthritis: a systematic review and meta-analysis

**DOI:** 10.3389/fimmu.2026.1816269

**Published:** 2026-07-17

**Authors:** Guangtao Li, Wenhui Xie, Jiaxi Liu, Yan Geng, Zhuoli Zhang

**Affiliations:** Department of Rheumatology and Clinical Immunology, Peking University First Hospital, Beijing, China

**Keywords:** meta-analysis, methotrexate, randomized controlled trials, rheumatoid arthritis, subcutaneous injections

## Abstract

**Objectives:**

To integrate evidence from randomized controlled trials (RCTs) comparing subcutaneous (SC) and oral methotrexate (MTX) for rheumatoid arthritis (RA) to provide optimal clinical treatment strategies.

**Methods:**

This meta-analysis conducted comprehensive search of PubMed, Web of Science, Embase and Cochrane Library, retrieving all relevant RCTs published up to June 18, 2025. Random-effects models were utilized to calculate the relative risk (RR), mean difference (MD) and their 95% confidence intervals (CIs), to evaluate efficacy and safety between subcutaneous injections and oral administration of MTX.

**Results:**

A total of 1,034 articles were retrieved, and 9 RCTs that met the criteria were ultimately included, involving 974 RA patients in total. In the primary random-effects analyses, compared to oral MTX, subcutaneous MTX increased the ACR20 response rate (RR = 1.15; 95%CI: 1.05, 1.25), increased the ACR50 response rate (RR = 1.14; 95%CI: 1.01, 1.29), and reduced the incidence of gastrointestinal (GI)-related adverse event (AE) (RR = 0.58; 95%CI: 0.40, 0.83) and diarrhea (RR = 0.42; 95%CI: 0.21, 0.84). Fixed-effect sensitivity analyses supported the ACR20 and GI-related safety findings, but the ACR50 result was attenuated. Subcutaneous MTX did not show statistically significant differences in ACR70 response rate, DAS28-ESR score, bioavailability area under the curve, C_max_, or the incidence of other AE.

**Conclusion:**

Compared with oral administration, subcutaneous MTX was associated with a higher ACR20 response and lower GI-related AE and diarrhea in the primary random-effects analyses. These findings suggested that subcutaneous MTX may be an effective and generally well-tolerated option, particularly for patients with inadequate response or poor gastrointestinal tolerability to oral MTX, while evidence for ACR50, ACR70, DAS28-ESR and bioavailability remains less certain.

## Highlights

- Subcutaneous MTX boosts RA’s ACR20/50 response rates.
- Subcutaneous MTX reduces RA’s GI-related AEs.
- Subcutaneous MTX may be considered when oral MTX response or tolerability is inadequate.

## 1 Introduction

Compared to traditional oral medication, subcutaneous methotrexate (MTX) has gained increasing attention. In psoriasis treatment, multiple guidelines and consensus recommendations have recommended subcutaneous injection of MTX as the preferred route over oral. For example, the 2020 EuroGuiDerm Guideline on the systemic treatment of Psoriasis vulgaris considered that MTX should be administered via subcutaneous injection as the preferred route, as it offers better safety and higher bioavailability (1). The 2023 Real-World Experience of MTX in the Treatment of Skin Diseases: an Italian Delphi Consensus also suggested that the preferred route of administration for MTX is subcutaneous injection (2). In the field of rheumatoid arthritis (RA) treatment, subcutaneous MTX has also gradually been included in the recommended list of guidelines. As recommended by the 2021 American College of Rheumatology Guideline for the Treatment of RA, for patients who are intolerant to oral MTX or whose treatment has not reached a target, switching to subcutaneous injection can be considered instead of replacing or adding other disease-modifying antirheumatic drugs (DMARDs) (3). The 2022 “Chinese Expert Consensus on Off-label Medication Use for Rheumatoid Arthritis” also suggested that for RA patients who are intolerant to oral MTX or whose treatment with the maximum tolerated dose via oral administration is not satisfactory, subcutaneous injection can be considered (4). However, the 2022 updated EULAR recommendations for the management of RA with synthetic and biological DMARDs first pointed out the working group had no specific preference regarding the administration route of MTX (5). The more recent 2025 update reaffirmed this recommendation. No unified consensus on the administration method of MTX has formed in RA.

Different administration methods may affect the therapeutic effect. MTX can be administered orally, by subcutaneous injection, intramuscular injection or intravenously. After oral administration, the absorption rate is relatively slow, and the peak concentration is reached approximately 1–2 hours later (6). Oral MTX needs to be absorbed under the action of reduced folate carrier protein (RFC) in the gastrointestinal (GI) tract. Some of the MTX is metabolized by gut microbiota and becomes inactive or is converted into glutamate in the liver (7). Thus, oral administration may lead to intestinal absorption saturation and nonlinear pharmacokinetics, characterized by lower bioavailability and pronounced inter-individual variability in some settings (8). Compared with oral MTX, subcutaneous administration has been reported to improve systemic exposure and tolerability in some studies, particularly at doses around or above 15 mg/week (9). For example, one standardized pharmacokinetic study reported that when 15 mg of MTX was administered by subcutaneous injection once, the maximum blood concentration (C_max_) was on average 0.86 μmol/L, which was 1.29 times that of the same dose taken orally (10). The same study reported higher bioavailability with subcutaneous administration, with larger differences at higher MTX doses (10).

Although subcutaneous injections of MTX show potential advantages in terms of pharmacokinetics and clinical efficacy, the relatively recent introduction of subcutaneous formulations has limited the available clinical evidence. Existing studies systematically comparing effectiveness of subcutaneous MTX and oral MTX reported inconsistent findings (11–13). Li et al. (11) synthesized evidence from two RCTs on clinical efficacy, reporting that SC MTX significantly improved ACR20, ACR70, and significantly reduce the incidence of nausea and diarrhea (11). In addition, Bujor et al. (13) pooled data from four RCTs and found that SC MTX improved ACR20 but did not significantly reduce the risk of adverse events. The study incorporated data from an abstract by Ahmed, which had substantial impact on clinical efficacy estimation (13). In contrast, a more recent study reported that SC MTX did not significantly improve ACR20, ACR50, or ACR70, nor did it reduce the risk of any adverse events (12). Differences in the number of studies included and the degree of potential heterogeneity likely contributed to the inconsistency in these results, despite broadly similar point estimates. On the other side, several randomized controlled trials (RCTs) and observational studies have compared the efficacy and safety of subcutaneous MTX administration with oral MTX administration (10, 14, 15). Two recent RCTs from China and Japan reported modest to substantial benefit of subcutaneous MTX over oral MTX (16, 17). To the best of our knowledge, few meta-analyses have systematically incorporated these studies, highlighting the need to update the existing evidence to ensure timely access to optimal treatment.

Herein, this study aims to conduct a systematic review and meta-analysis to integrate the RCT evidence of subcutaneous MTX and oral MTX in RA patients and comprehensively evaluate both efficacy and safety, enhancing the current evidence level and providing more valuable evidence-based support for clinical practice.

## 2 Methods

### 2.1 Search strategy

This systematic review and meta-analysis were conducted according to the PRISMA (Preferred Reporting Items for Systematic Reviews and Meta-analyses) guidelines. An artificial search from PubMed, Cochrane Library, Web of Science and Embase databases was performed until 18 June 2025. The detailed search strategy for each of the 4 databases was shown in **Supplementary Material 1**. The search key words are: ((Methotrexate) OR (MTX)) AND (rheumatoid arthritis) AND ((subcutaneous) OR (parenteral) OR (injectable) OR (intramuscular)) AND ((oral) OR (tablet)) AND (clinical trial).

### 2.2 Study selection

The imported literature was checked and selected through two steps. First, according to the population, intervention, comparator, outcomes, study design (PICOS) principle, the article titles and abstracts were screened. Next, a full-text search on the selected articles and based on the exclusion criteria was conducted, eliminating those do not meet the requirements. Literature screening and basic data extraction were independently performed by three researchers following the established inclusion and exclusion criteria. In case of disagreement, discrepancies were resolved through discussion and cross-verification.

### 2.3 Inclusion and exclusion criteria

The inclusion and exclusion criteria for literature screening were as follows. Inclusion criteria: (1) RCTs or randomized crossover studies conducted in adults diagnosed with RA. (2) Studies comparing oral administration with subcutaneous, parenteral, or intramuscular injection of MTX, with no restrictions on MTX dosage; concomitant medications are allowed. (3) Studies must include one of the following outcomes: American college of Rheumatology (ACR) response criteria (ACR20/50/70 response), 28-joint Disease Activity Score for erythrocyte sedimentation rate (DAS28-ESR), Simplified Disease Activity Index - Low Disease Activity (SDAI-LDA), adverse event (AE), area under the curve (AUC_0-t_), maximum (peak) plasma concentration(C_max_), and time to reach C_max_(T_max_). Exclusion criteria: (1) Patients with autoimmune diseases other than RA. (2) observational and retrospective studies. (3) the same research that has been published repeatedly.

### 2.4 Bias assessment

The assessment of study bias was conducted by two independent reviewers. In case of any disagreement, discrepancy was resolved through consensus negotiation. The risk of bias was assessed using the Risk of Bias 2 (ROB2) tool (18). This scoring tool encompasses five domains: bias due to randomization process, bias due to deviation from the intended intervention, bias due to missing data, bias due to outcome measurement, and bias due to selective reporting. The risk of bias in each domain is assessed as low, high, or of some concern. Scores were given separately for the random parallel controlled trials and the randomized crossover trials.

### 2.5 Evaluation of evidence uncertainty

The evidence level was evaluated utilizing the Grading of Recommendations Assessment, Development and Evaluation (GRADE) framework (19). This framework classifies the evidence levels as very low, low, medium and high. The evidence level of RCTs was rated starting from high and then was downgraded according to the rules. The evidence level can be downgraded in the following aspects: (1) risk of bias, if more than 25% of the studies have a high risk of bias, it indicates that the research results are likely to have bias; (2) inconsistency of research results, when using I^2^ statistic to evaluate the consistency of the research results, the larger I^2^ value (such as I^2^ ≥ 50%) indicates that the research results are more likely to have significant heterogeneity; (3) indirectness, assessing the extent to which the existing evidence directly addresses the clinical problem, such as whether there are factors that limit the generalization ability of the results; (4) imprecision, such as a wide 95% confidence interval (CI) or the 95%CI includes the minimum clinically significant difference; (5) publication bias.

### 2.6 Data extraction

Two researchers independently screened, extracted and cross-checked the literature, and those with different opinions were resolved through consensus negotiation. The extracted data included: (1) author, publication date, country, demographic data of the subjects (age and gender), treatments (duration, dosage, etc.) in experimental and control groups, sample size in each group; (2) number of participants in each group who experienced events (ACR20/50/70) at the 4th/8th/12th/24th weeks where reported and extractable, average value (standard deviation) of the change in DAS28-ESR from baseline in the experimental group at the 4th/8th/12th/24th weeks where reported and extractable; (3) number of (any) AE in each group, and (4) bioavailability assessment values (AUC_0-t_, C_max_, T_max_) in each group.

### 2.7 Statistical analysis

This study employed R version 4.4.2 for data analysis. Meta-analysis was conducted using the “meta” and “metafor” R packages. In this study, the data of ACR20, ACR50, ACR70, and DAS28-ESR were combined separately, and the data of any AE, TEAE, and each type of AE were also combined separately. For the outcome indicators ACR20/50/70, the efficacy was evaluated using relative risk (RR) (20, 21); for the outcome indicator DAS28-ESR, the efficacy was evaluated using mean difference (MD); for the bioavailability indicators, the AUC from administration to the last measured concentration, C_max_, were evaluated using MD for bioavailability; for AE, safety was evaluated using RR.

Due to clinical heterogeneity was expected across studies in patient populations, MTX dose, treatment background, assessment time point, geographic region, and route/formulation, a random-effects model was used as the primary model for all outcomes, regardless of the observed I2 value. This approach was chosen because a low I2 does not exclude clinically important between-study differences. To assess model dependence for outcomes with low statistical heterogeneity, fixed-effect sensitivity analyses were also calculated for outcomes with I2 <50% and compared with the primary random-effects estimates. The RR and MD of the integrated data were estimated along with their 95%CIs (22), and forest plots were constructed to present the integrated RR and MD. Differences among groups were tested using the Z-test, and a P-value less than 0.05 indicated a statistically significant difference. The heterogeneity of the studies was evaluated by the Q test and the I2 statistics (23). An I2 value of 0-25% indicated low heterogeneity, 25-50% indicated moderate heterogeneity, 50-75% indicated high heterogeneity, and 75-100% indicated very high heterogeneity. Sensitivity analysis was conducted through the leave-one-out method, each included study was excluded one by one to assess the stability of the effect values (RR and MD). The funnel plot and Egger test were used to assess publication bias (24).

Because assessment time point, geographic region and treatment background could plausibly modify treatment effects, we performed *post hoc* exploratory subgroup analyses for ACR20/50/70 by endpoint time point (12 vs. 24 weeks) and geographic region when data were available. We also tabulated the availability of 4-, 8-, 12-, and 24-week ACR data to clarify why formal pooled analyses at 4 and 8 weeks were not feasible. These analyses were considered descriptive because several strata contained only one or two studies and early time-point event counts were not consistently extractable across trials.

## 3 Results

### 3.1 Search results

**Figure 1:**
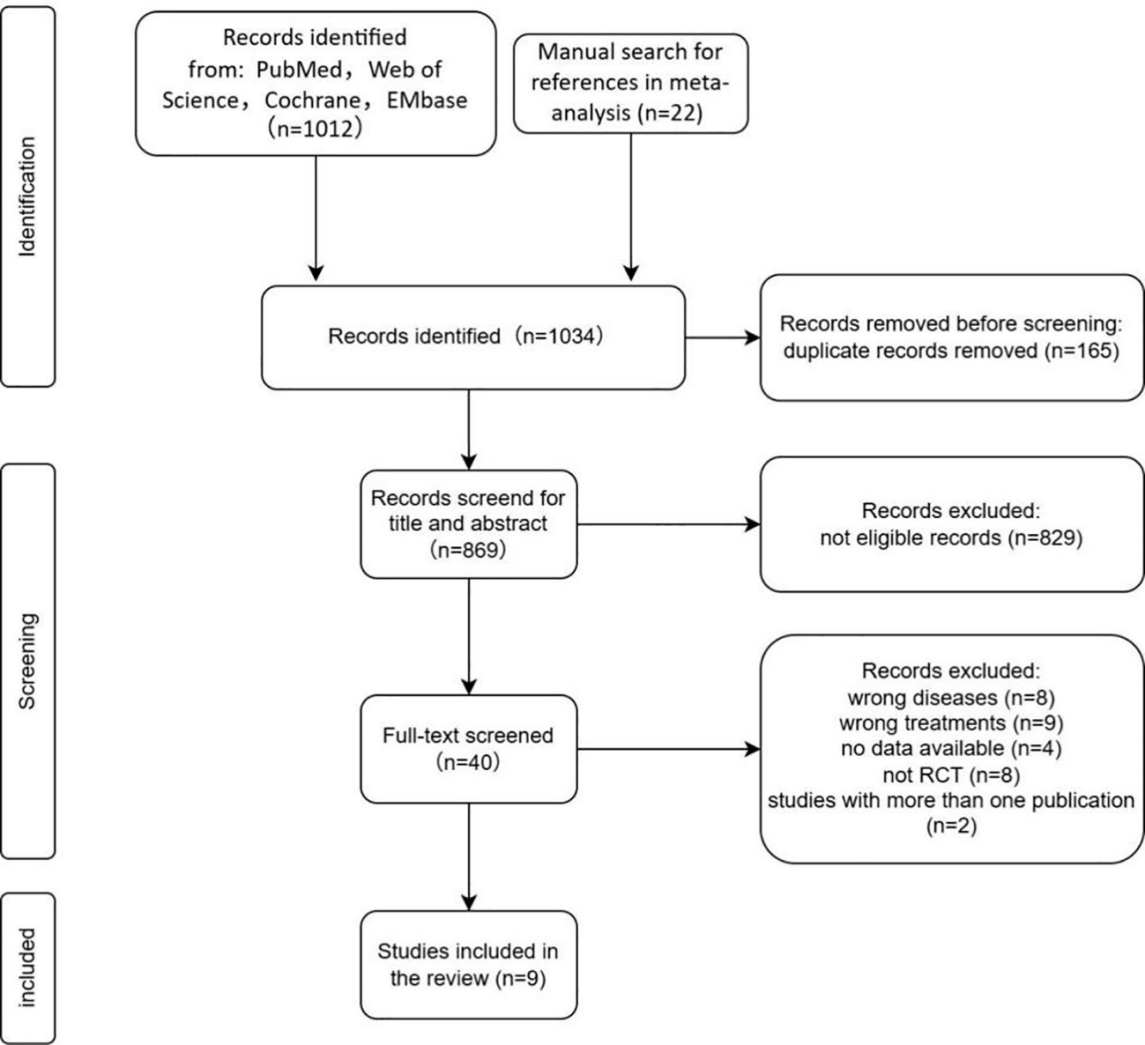
PRISMA flow diagram of the literature search and study selection process.

A total of 1,034 articles were retrieved from 4 databases. Subsequently, 165 repetitive articles and 829 articles not related to the topic based on titles and abstracts were excluded. Among the rest 40 articles that were fully reviewed, 8 did not meet the target indications, 9 did not conform to the treatment methods, 4 lacked target data, 8 were not RCTs, and 2 were duplicate studies. Finally, 9 articles were eligible for further analysis. The detailed screening process was shown in **Figure 1**.

### 3.2 Study characteristics

The basic characteristics of the included studies were presented in **Supplementary Table 1**. A total of 9 clinical trials, involving 974 patients, were included (10, 14, 16, 17, 25–29). 74% of the patients were female, with an average age of approximately 50 years. The average duration of RA was 0.91 to 159 months. The dose of MTX was 2.5–25 mg/week, and the MTX treatment course was 8–24 weeks. Among them 5 studies evaluated safety and effectiveness (16, 17, 25, 27, 28), 2 studies evaluated bioavailability and safety (10, 14), 2 studies evaluated bioavailability (26, 29). The MTX dosage ranged from 7.5 mg/week to 25 mg/week, with 15 mg/week being the most commonly used. The treatment duration ranged from 8 to 24 weeks, with a 12-week regimen being the most frequently applied.

To clarify MTX monotherapy versus background therapy and center setting, **Supplementary Table 1** was expanded to include MTX route and dose, concomitant RA therapy, country/region, center setting and assessment windows. The included efficacy trials were not uniformly strict MTX monotherapy: Braun et al. and Qiao et al. did not allow concomitant DMARDs during the randomized comparison but permitted stable NSAIDs and/or glucocorticoids; Tanaka et al. administered folic acid; Dhaon et al. used hydroxychloroquine and short-course methylprednisolone as background therapy; and Islam et al. did not report sufficient background-therapy details in the available report. Multicentre randomized trials included Qiao et al., Braun et al. and Tanaka et al.; Schiff et al. was a multicentre pharmacokinetic crossover study.

### 3.3 Clinical efficacy

**Figure 2:**
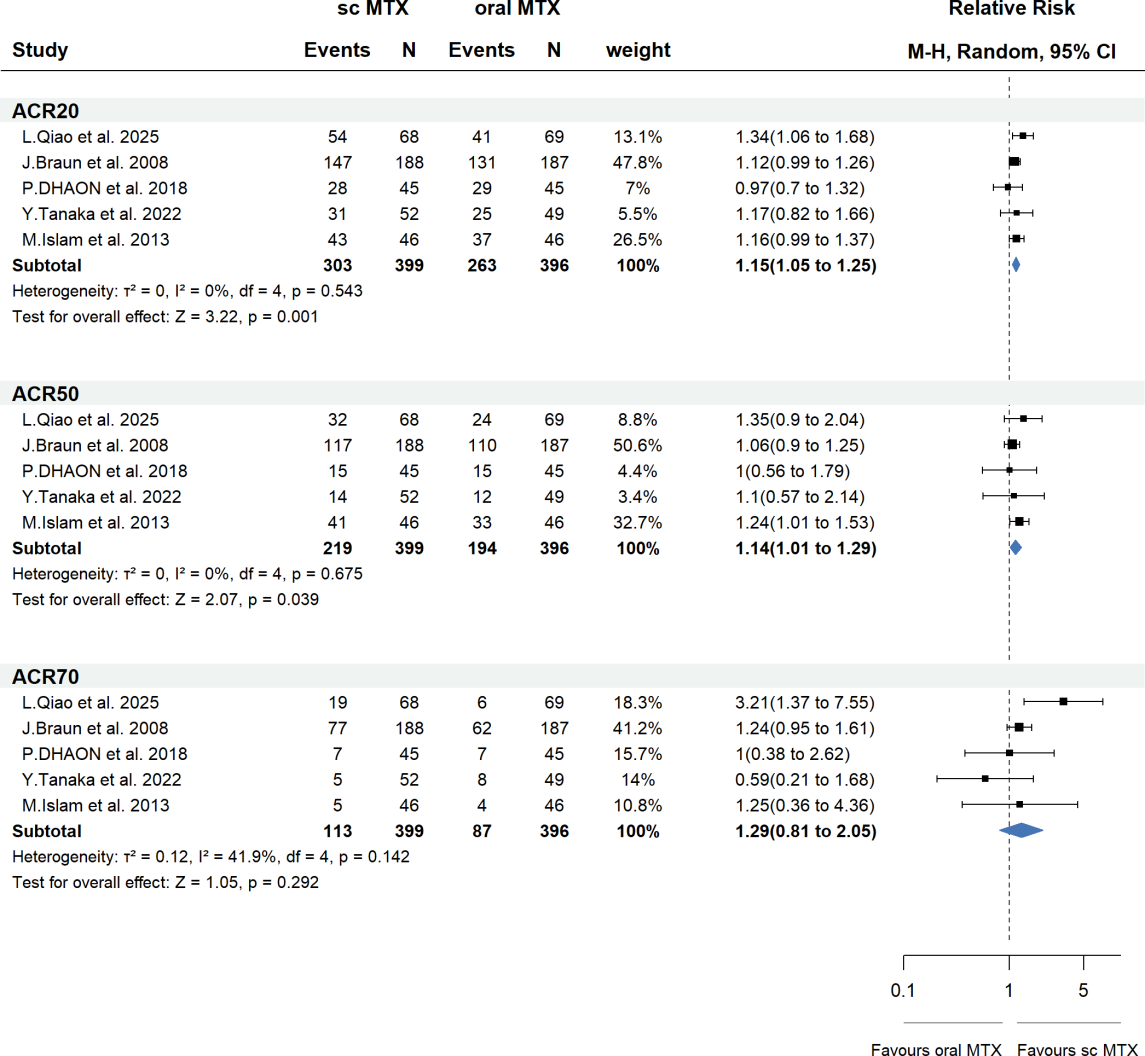
Forest plot comparing ACR20, ACR50, and ACR70 response rates between SC and oral MTX in RA patients.

**Figure 3:**
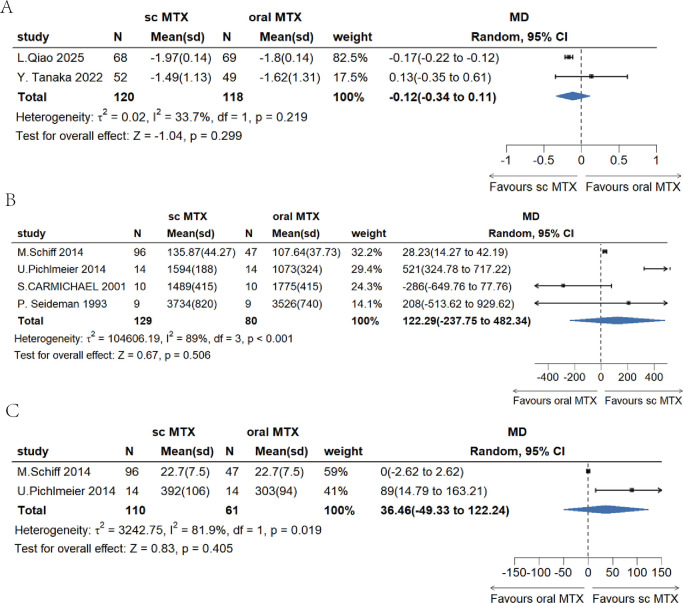
Forest plots comparing continuous outcomes between SC MTX and oral MTX in RA patients. **(A)** 28-joint disease activity score for erythrocyte sedimentation rate (DAS28-ESR); **(B)** area under the concentration-time curve from administration to the last measured concentration (AUC_0-t_); **(C)** maximum plasma concentration (C_max_).

A total of 5 articles were included in the efficacy analysis based on ACR20, ACR50 and ACR70, and 2 articles in the analysis based on DAS28-ESR (**Figures 2**, **3A**). In the primary random-effects analyses, compared with oral MTX, subcutaneous MTX demonstrated a significant improvement in ACR20 response rate (RR = 1.15, 95%CI: 1.05, 1.25) and ACR50 response rate (RR = 1.14; 95%CI: 1.01, 1.29), although the ACR50 result was attenuated in the fixed-effect sensitivity analysis (**Supplementary Table 2**). However, in higher-standard response indicators, the improvement in ACR70 (RR = 1.29, 95%CI: 0.81, 2.05) with subcutaneous injection did not reach statistical significance, as the 95% confidence interval included 1. This may suggest that current evidence is insufficient to confirm its superiority in moderate to high-level disease improvement. In terms of disease activity score (DAS28-ESR), there was no statistically significant difference between subcutaneous and oral MTX in the primary random-effects analysis (MD=-0.12, 95% CI: -0.34, 0.11). Regarding heterogeneity, the results for ACR20/50 and DAS28-ESR showed good consistency (I2 = 0%-33.7%), while ACR70 showed moderate heterogeneity (I2 = 41.9%).

Exploratory subgroup analyses by assessment time point and geographic region are presented in **Supplementary Table 3**. For ACR20, the estimates were directionally similar at 12 weeks (RR = 1.28, 95%CI: 1.06-1.56) and 24 weeks (RR = 1.12, 95%CI: 1.02-1.22), and in Asian studies (RR = 1.17, 95%CI: 1.05-1.32) and the single European efficacy RCT (RR = 1.12, 95%CI: 0.99-1.26). ACR50 and ACR70 subgroup estimates were less precise, especially for ACR70 at 12 weeks. The feasibility of 4-, 8-, 12-, and 24-week analyses is summarized in **Supplementary Table 4**. Exact 4- and 8-week arm-level ACR20/50/70 data were completely extractable only from Tanaka et al., while early data from Qiao et al. were partly descriptive or figure-based and Braun et al. did not provide exact extractable early arm-level counts. Therefore, we did not conduct formal 4- or 8-week pooled analyses. Because most subgroup strata contained one to three studies and the Americas/Oceania contributed bioavailability rather than ACR efficacy RCTs, these findings should be interpreted as descriptive rather than definitive evidence of regional or ethnic differences.

Dose-specific evidence and route-focused sensitivity analyses are summarized in **Supplementary Table 5**. Evidence below 15 mg/week was limited to one randomized clinical comparison (Tanaka et al.; SC MTX 7.5 mg/week vs oral MTX 8 mg/week), and the ACR20/50/70 estimates were imprecise. Therefore, the current evidence does not support a formal <15 mg/week subgroup meta-analysis and should not be interpreted as proving a pharmacokinetic or clinical advantage of SC/parenteral MTX at doses below 15 mg/week.

After excluding the efficacy trial with an IM/parenteral MTX arm (Dhaon et al.), the pooled estimates remained similar for ACR20 (RR = 1.16, 95%CI: 1.06-1.27) and ACR50 (RR = 1.14, 95%CI: 1.01-1.29), while ACR70 remained non-significant (RR = 1.35, 95%CI: 0.76-2.41). These findings suggest that the main ACR20/50 conclusions were not driven by the IM/parenteral trial, although route heterogeneity remains an important limitation.

### 3.4 Bioavailability

Regarding bioavailability, a total of 4 articles were included in the analysis based on AUC_0-t_ (**Figure 3B**). The results showed that compared with oral administration, subcutaneous MTX did not significantly increase the AUC_0-t_ (MD = 122.62, 95%CI: -237.28, 482.52). The analysis based on C_max_ included 2 articles (**Figure 3C**), and no statistical difference in C_max_ was observed between subcutaneous and oral MTX (MD = 36.46, 95%CI: -49.33, 122.24) (**Supplementary Figure 1**).

When the AUC analysis was restricted to the two SC-vs-oral studies (Schiff et al. and Pichlmeier et al.), the pooled estimate remained imprecise (MD = 264.50, 95%CI: -218.01 to 747.00; I2 = 95.9%). The C_max_ analysis already included only SC-vs-oral studies and remained non-significant (MD = 36.46, 95%CI: -49.33 to 122.24). Thus, the available PK evidence is compatible with higher exposure after SC MTX in some settings but is not sufficient to confirm a pooled low-dose advantage below 15 mg/week.

### 3.5 Safety

**Figure 4:**
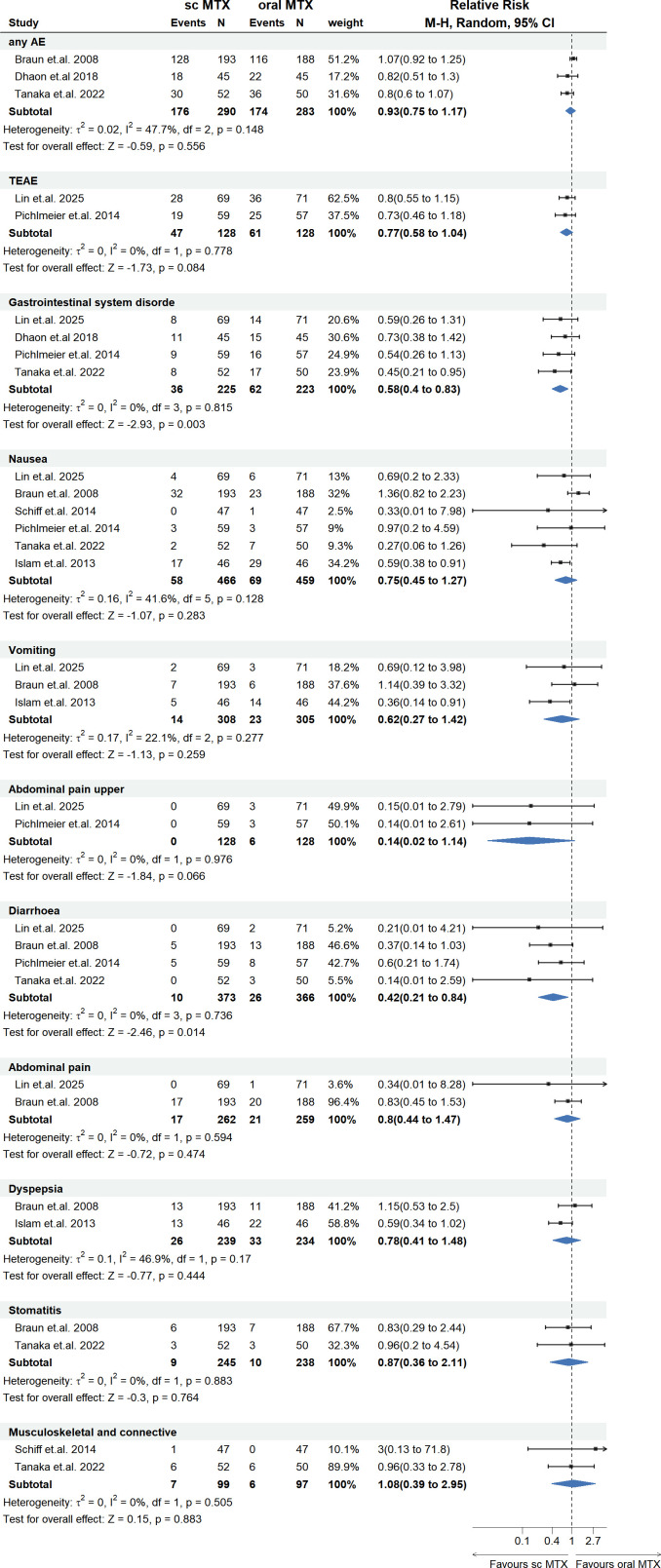
Forest plot comparing adverse events between SC MTX and oral MTX in RA patients. AE, adverse event; TEAE, treatment-emergent adverse event; GI, gastrointestinal; AP, abdominal pain; APU, abdominal pain upper; MC, musculoskeletal and connective tissue disorders.

Regarding the safety analysis (**Figure 4**), compared with oral administration, subcutaneous MTX significantly reduced GI AE (RR = 0.58, 95%CI: 0.4, 0.83), especially diarrhea (RR = 0.42, 95%CI: 0.21, 0.84), and the study heterogeneity was low (I2≈0%). The subcutaneous MTX did not significantly reduce other pooled AE categories, but overall, it showed a trend that the subcutaneous MTX could reduce the incidence of AE. Clinically important MTX toxicities, including hepatic injury, renal impairment, myelosuppression and pulmonary toxicity, were reviewed but not pooled because they were sparsely and inconsistently reported, used heterogeneous definitions, or lacked extractable arm-level route-comparison data; the reasons are summarized in **Supplementary Table 6**.

### 3.6 Quality assessment

The evidence certainty for the efficacy and safety integration results was assessed via the GRADE tool. According to **Supplementary Table 7**, the subcutaneous MTX could significantly improve the ACR20 score and significantly reduce GI AE and diarrhea, all supported by a high level of evidence.

**Figure 5:**
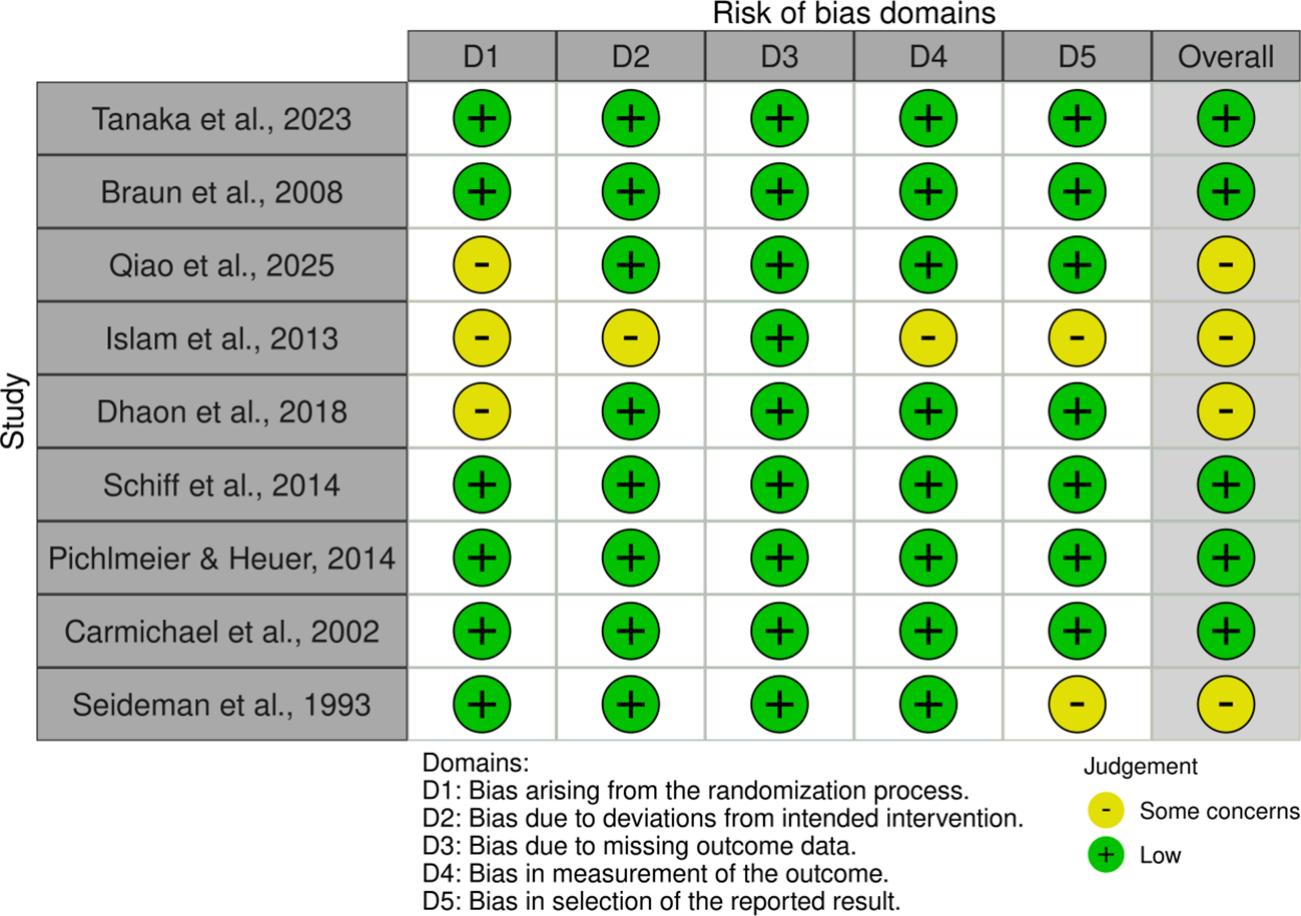
Risk-of-bias assessment for individual included studies using the ROB2 tool.

**Figure 6:**
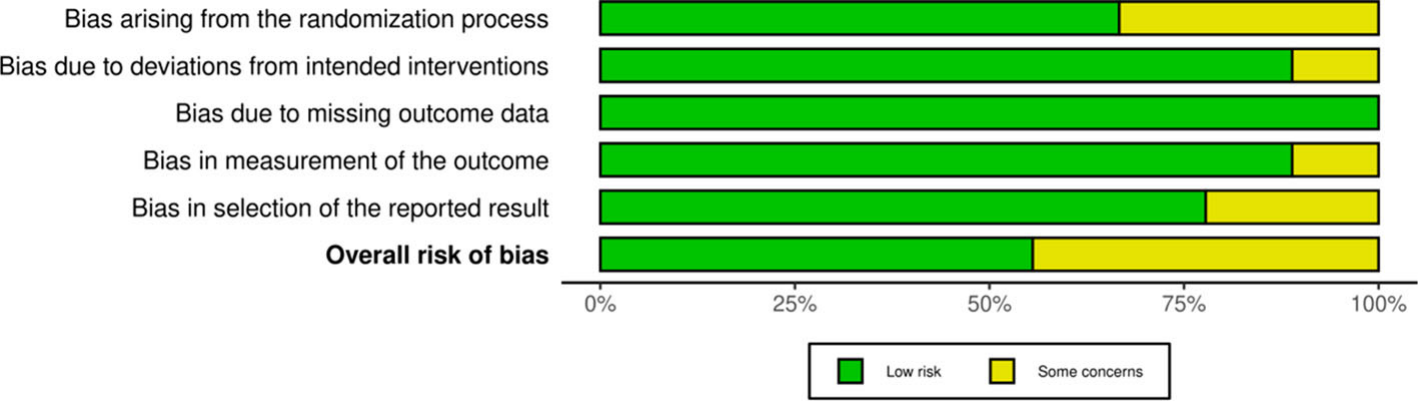
Summary of risk-of-bias assessments across included studies using the ROB2 tool.

The risk of bias assessment for the included articles was shown in **Figures 5** and **6**. Overall, there were 5 studies with low risk of bias, while 4 studies had potential bias. Among them, the bias mainly concentrated on the random process aspect, accounting for approximately 33%, and it was the primary source of bias.

The funnel plot (**Supplementary Figures 2-S4**) and the Egger test (**Supplementary Table 8**) showed that all the funnel plots for the outcomes present good symmetry, and the Egger test did not reach a statistically significant level in any case, indicating that there is no clear evidence suggesting publication bias.

### 3.7 Sensitivity analysis

The results of sensitivity analysis clearly showed that the point and interval estimate of effectiveness on ACR20 were consistent across all studies. However, the results of Qiao et al. and Islam et al. have an impact on the effective analysis based on ACR50 (**Supplementary Figure 5**). After excluding these two studies individually, the RR values were 1.12 (95%CI: 0.98-1.27, *P* = 0.0861) and 1.09 (95%CI:0.94-1.26, *P* = 0.2463), respectively, with the point estimates changing only slightly, although the 95%CI did not reach statistical significance. After adding the Braun et al. week-24 ACR70 data, the efficacy analysis of ACR70 and its leave-one-out sensitivity analyses remained statistically non-significant (**Supplementary Figure 5**). The efficacy analysis of DAS28 showed that in Qiao et al.’s study, the subcutaneous MTX significantly increased DAS28 (**Supplementary Figure 6**). However, in Tanaka et al.’s study, the subcutaneous MTX did not significantly improve DAS28 (**Supplementary Figure 6**). Regarding the safety analysis, the subcutaneous MTX significantly reduced the incidence of GI (**Supplementary Figure 7**). The research results of Braun et al. have a significant impact on the safety analysis based on diarrhea. After excluding this study, the RR was 0.47 (95%CI: 0.18-1.2, *P* = 0.1134) (**Supplementary Figure 7**).

Fixed-effect sensitivity analyses for outcomes with I2 <50% are summarized in **Supplementary Table 6**. Most inferences were consistent with the primary random-effects analyses, including ACR20, GI adverse events and diarrhea. ACR50 was attenuated under the fixed-effect model (RR = 1.12, 95%CI: 0.99-1.28; P = 0.0717), and DAS28-ESR reached statistical significance under the fixed-effect model (MD=-0.17, 95%CI: -0.22 to -0.12; P<0.001) despite the non-significant primary random-effects estimate. ACR70 remained non-significant under the primary random-effects model and was borderline under fixed-effect sensitivity (RR = 1.26, 95%CI: 0.999-1.599; P = 0.0508). Therefore, the random-effects inference was retained as primary, and ACR50, ACR70 and DAS28-ESR should be interpreted cautiously.

## 4 Discussion

This study conducted a meta-analysis to integrate the data from RCTs, comparing the efficacy, bioavailability and safety of oral MTX and subcutaneous MTX in patients with RA. A total of 9 RCTs were included, and the primary random-effects analyses showed that subcutaneous MTX increased the ACR20/50 response rate and significantly reduced the incidence of GI AE, especially diarrhea. The results also showed a non-significant trend of improving ACR70, reducing DAS28-ESR, and increasing AUC_0-t_ and C_max_. This study provided updated evidence for the efficacy and gastrointestinal tolerability of subcutaneous MTX, although ACR50 should be interpreted cautiously because it was attenuated in the fixed-effect sensitivity analysis. Compared to previous meta-analyses, our study included two latest East Asian RCTs (16, 17), enriching ethnic diversity and strengthening generalizability.

Our findings on clinical efficacy align with two key meta-analyses. Bujor et al. (13) reported a significant increase in ACR20 response with subcutaneous MTX (OR = 3.02, 95%CI:1.41,6.46) in 4 RCTs (13), and Li et al. (11) observed similar findings for ACR20 (OR = 1.68, 95%CI: 1.09, 2.61) and ACR70 (OR = 1.52, 95%CI: 1.02, 2.26) in 2 RCTs (11)—consistent with our ACR20 result (RR = 1.15, 95%CI:1.05,1.25). Additionally, our findings on clinical efficacy are consistent with multiple large-scale cohort studies (28–33). Moura et al. (30) showed that compared with monotherapy with oral MTX, subcutaneous MTX treatment was more durable (time to treatment change, adjusted HR = 0.52, 95%CI: 0.41, 0.66) and had a lower rate of drug switching (45% vs 79%, HR = 0.52, 95%CI: 0.40, 0.67), indicating that subcutaneous MTX can reduce the failure rate of treatment and improve clinical efficacy (30). Similarly, Wan et al. (34) compared the effects of increasing the oral MTX dose and the oral-to-subcutaneous MTX transfer strategy on the timing of initiating biologic disease-modifying antirheumatic drugs (bDMARDs) (34). The results showed that the patients who switched to subcutaneous injection started bDMARDs significantly later by about 9 months, indicating that subcutaneous MTX has a better effect on disease control. The same finding was concluded from the cohort study by Gottheil et al. (35), showing that subcutaneous MTX could significantly delay the time of first use of biologics (HR = 0.47, *P* = 0.015) (35). However, Wang et al. (12) found no significant difference in ACR20 between oral and subcutaneous MTX (OR = 0.68, 95%CI:0.4,1.15) (12). The inconsistent result may stem from their exclusion of recent evidence (e.g., Qiao et al.2025) (16).

Our findings on safety are consistent with a previous meta-analysis (11), demonstrating subcutaneous MTX can significantly reduce diarrhea and other GI AE. In addition, several observational studies supported our findings (36, 37). Bakker et al.’s (38) showed that subcutaneous MTX demonstrated a higher efficacy, especially for those patients with poor absorption or GI problems affecting treatment (38). Qiao et al. (16) suggested that the safety profile of subcutaneous MTX was similar to that of oral MTX in general, and the incidence, occurrences and preferred term types of drug-related TEAE of GI system disorders were lower. However, the current RCT evidence was not sufficient to compare rare or clinically important toxicities such as hepatic injury, renal impairment, myelosuppression or pulmonary toxicity by administration route, and these outcomes should be monitored in future trials with standardized definitions.

Notably, our meta-analysis found no significant differences in AUC_0-t_ (MD = 122.62,95%CI: -237.28,482.52) or C_max_ (MD = 36.46, 95%CI: -49.33,122.24) between subcutaneous and oral MTX, despite a trend toward higher bioavailability in subcutaneous administration. This contrasts with Pichlmeier et al. (10), who reported 35% higher bioavailability with subcutaneous MTX. The discrepancy may be attributed to several factors. First, our analysis included a wider dose range (2.5–25 mg/week) and more heterogeneous study populations, whereas Pichlmeier et al. used standardized doses. Dose difference and interindividual difference in MTX absorption may lead to variation in bioavailability across studies. Second, four studies with a total of 129 patients were included for analysis of bioavailability, among which two studies were conducted over 20 years ago. Differences in protocols and analytical methods in these studies may occur over time and may have contributed to the variation of estimates of AUC_0-t_ and C_max._

Although there is potential heterogeneity in the assessment time of efficacy endpoint, the primary ACR20 and GI-related safety findings were generally robust. To account for potential heterogeneity, our study employed a random-effects model for pooling results. The heterogeneity test results showed that the overall level of heterogeneity is relatively low for most outcomes (ACR20: I^2^ = 0%, ACR50: I^2^ = 0%, DAS28-ESR: I^2^ = 33.7%), with only ACR70 showing moderate heterogeneity (I^2^ = 41.9%). Fixed-effect sensitivity analyses further supported the robustness of ACR20 and GI-related safety findings, whereas ACR50, ACR70 and DAS28-ESR required cautious interpretation. In addition, a study by Wang.Y, et al. (39) also showed that short-term efficacy reliably predicts long-term efficacy, and the treatment effect at month 3 approaches a “plateau” (39). This study implies small to no heterogeneity of efficacy in week 12 and week 24, supporting the comparability of week 12 and week 24 assessments. Furthermore, although patients included in our study have varying disease duration, studies conducted by Majorczyk et al. (40) and Drouin and Haraoui (41) suggested that no significant correlation between disease duration and the efficacy of MTX. Therefore, including patients at different disease stages in our study may not affect the robustness of results.

Although its advantages, our study has several limitations. First, MTX doses varied (2.5–25mg/week) across included studies, with differences in initial/maintenance doses and adjustment strategies (42). Due to the small number of included studies (n=9), subgroup analyses by dose were not feasible. Despite Rubio-Romero et al. (43) confirmed comparable efficacy between common starting doses (7.5mg vs.15mg) (43), variations in clinical efficacy and bioavailability may still occur particularly in low (2.5mg/week) and high (>15mg/week) dose groups. Second, since most of the studies included in our analysis did not provide DAS28-ESR data, the evidence on whether subcutaneous MTX improves DAS28-ESR compared to oral MTX is unclear. Although many studies have supported that subcutaneous MTX can improve DAS28 (44–46), high-level evidence is still lacking. Lastly, since only one study included in our analysis used DAS28-CRP as the primary efficacy endpoint, no pooled evidence showing effectiveness of subcutaneous MTX on improvement of DAS28-CRP was provided in our analysis. In addition, concomitant medications and treatment context varied across studies and were incompletely reported in Islam et al., limiting confirmatory subgroup analyses by MTX monotherapy versus combination or background therapy. Route heterogeneity also needs to be considered, because one efficacy study used IM/parenteral MTX and some bioavailability studies included IV or IM route comparisons rather than strict SC-vs-oral comparisons.

Future research could be explored in several directions. First, long-term RCTs (≥52 weeks) are needed to assess hard endpoints, including sustained DAS28 remission (<2.6), radiographic progression, and treatment persistence. Second, subgroup analyses should be performed according to disease stage (early vs. established RA), concomitant DMARD use, age (≥65 vs. <65years), dose (>15mg/week vs. ≤15mg/week), therapeutic strategies (treatment-to-target, T2T vs. Non-T2T) to inform personalized strategies. Third, mechanistic studies linking subcutaneous MTX to intracellular MTX polyglutamate accumulation (e.g., RBC MTXGlu) (45) are needed to explain underlying efficacy mechanisms. Fourth, due to the potential difference between DAS28-ESR and DAS28-CRP (47, 48), future studies should directly compare the effect of subcutaneous and oral MTX using both efficacy endpoints. Fifth, our results imply that bioavailability differences may be dose dependent. Thus dose-stratified pharmacokinetic studies are needed to confirm the causal association between doses and bioavailability. Lastly, real-world studies are needed to validate our findings in diverse populations and enhance their generalizability, particularly in under representative regions.

Recent mechanistic work further suggests that MTX polyglutamates accumulate in peripheral blood monocytes within days and that MTX modulates monocyte transcriptional signatures after repeated dosing; non-classical monocyte-associated genes including MAF, FCGR3B and ICAM4 may predict clinical response (49). This supports biomarker-oriented research, but because that study did not compare administration routes, it should be viewed as a mechanistic hypothesis rather than evidence that SC and oral MTX differ through this pathway.

## 5 Conclusion

This study compared the efficacy and safety of oral MTX and subcutaneous MTX. The results showed that subcutaneous MTX significantly increased the ACR20 response rate by 12–24 weeks and significantly reduced the incidence of GI AE, showing evidence of improved ACR20 response and better gastrointestinal tolerability. These findings support SC MTX as an effective alternative to oral MTX, particularly for patients with inadequate response or poor gastrointestinal tolerability to oral MTX. Further long-term and dose-stratified studies are warranted.

## Data Availability

The raw data supporting the conclusions of this article will be made available by the authors, without undue reservation.
