# Supplementary material for: Subcutaneous methotrexate compared with oral methotrexate in rheumatoid arthritis: a systematic review and meta-analysis
Source: Front Immunol. 2026 Jul 17;17:1816269. doi: 10.3389/fimmu.2026.1816269 (PMC13424238; doi:10.3389/fimmu.2026.1816269)
Supplement: Supplementary file 1 [file DataSheet1.docx]

**The search strategy for each of the 4 databases**

**PubMed: 85**

Search: ((Methotrexate) OR (MTX)) AND (rheumatoid arthritis) AND (subcutaneous OR parenteral OR injectable OR intramuscular) AND ((oral) OR (tablet)) AND (clinical trial) Filters: Abstract, Full text, Humans, Exclude preprints

(("methotrexate"[Supplementary Concept] OR "methotrexate"[All Fields] OR "methotrexate"[MeSH Terms] OR "methotrexate s"[All Fields] OR "methotrexates"[All Fields] OR "MTX"[All Fields]) AND ("arthritis, rheumatoid"[MeSH Terms] OR ("arthritis"[All Fields] AND "rheumatoid"[All Fields]) OR "rheumatoid arthritis"[All Fields] OR ("rheumatoid"[All Fields] AND "arthritis"[All Fields])) AND ("subcutaneous"[All Fields] OR "subcutaneously"[All Fields] OR "subcutanous"[All Fields] OR ("parenteral nutrition"[MeSH Terms] OR ("parenteral"[All Fields] AND "nutrition"[All Fields]) OR "parenteral nutrition"[All Fields] OR "parenteral"[All Fields] OR "parenterally"[All Fields] OR "parenterals"[All Fields]) OR ("inject"[All Fields] OR "injectability"[All Fields] OR "injectant"[All Fields] OR "injectants"[All Fields] OR "injectate"[All Fields] OR "injectates"[All Fields] OR "injected"[All Fields] OR "injectible"[All Fields] OR "injectibles"[All Fields] OR "injecting"[All Fields] OR "injections"[MeSH Terms] OR "injections"[All Fields] OR "injectable"[All Fields] OR "injectables"[All Fields] OR "injection"[All Fields] OR "injects"[All Fields]) OR "intramuscular"[All Fields]) AND ("mouth"[MeSH Terms] OR "mouth"[All Fields] OR "oral"[All Fields] OR ("tablet s"[All Fields] OR "tabletability"[All Fields] OR "tableted"[All Fields] OR "tablets"[Supplementary Concept] OR "tablets"[All Fields] OR "tablet"[All Fields] OR "tablets"[MeSH Terms] OR "tableting"[All Fields] OR "tabletted"[All Fields] OR "tabletting"[All Fields])) AND ("clinical trial"[Publication Type] OR "clinical trials as topic"[MeSH Terms] OR "clinical trial"[All Fields])) AND ((excludepreprints[Filter]) AND (fha[Filter]) AND (fft[Filter]) AND (humans[Filter]))

Translations

Methotrexate: "methotrexate"[Supplementary Concept] OR "methotrexate"[All Fields] OR "methotrexate"[MeSH Terms] OR "methotrexate's"[All Fields] OR "methotrexates"[All Fields]

rheumatoid arthritis: "arthritis, rheumatoid"[MeSH Terms] OR ("arthritis"[All Fields] AND "rheumatoid"[All Fields]) OR "rheumatoid arthritis"[All Fields] OR ("rheumatoid"[All Fields] AND "arthritis"[All Fields])

subcutaneous: "subcutaneous"[All Fields] OR "subcutaneously"[All Fields] OR "subcutanous"[All Fields]

parenteral: "parenteral nutrition"[MeSH Terms] OR ("parenteral"[All Fields] AND "nutrition"[All Fields]) OR "parenteral nutrition"[All Fields] OR "parenteral"[All Fields] OR "parenterally"[All Fields] OR "parenterals"[All Fields]

injectable: "inject"[All Fields] OR "injectability"[All Fields] OR "injectant"[All Fields] OR "injectants"[All Fields] OR "injectate"[All Fields] OR "injectates"[All Fields] OR "injected"[All Fields] OR "injectible"[All Fields] OR "injectibles"[All Fields] OR "injecting"[All Fields] OR "injections"[MeSH Terms] OR "injections"[All Fields] OR "injectable"[All Fields] OR "injectables"[All Fields] OR "injection"[All Fields] OR "injects"[All Fields]

oral: "mouth"[MeSH Terms] OR "mouth"[All Fields] OR "oral"[All Fields]

tablet: "tablet's"[All Fields] OR "tabletability"[All Fields] OR "tableted"[All Fields] OR "tablets"[Supplementary Concept] OR "tablets"[All Fields] OR "tablet"[All Fields] OR "tablets"[MeSH Terms] OR "tableting"[All Fields] OR "tabletted"[All Fields] OR "tabletting"[All Fields]

clinical trial: "clinical trial"[Publication Type] OR "clinical trials as topic"[MeSH Terms] OR "clinical trial"[All Fields]

**Embase: 428**

('methotrexate'/exp OR methotrexate OR 'mtx'/exp OR mtx) AND ('rheumatoid arthritis'/exp OR 'rheumatoid arthritis' OR (rheumatoid AND ('arthritis'/exp OR arthritis))) AND (subcutaneous OR parenteral OR 'injectable'/exp OR injectable OR intramuscular) AND (oral OR 'tablet'/exp OR tablet) AND ('clinical trial'/exp OR 'clinical trial' OR (('clinical'/exp OR clinical) AND ('trial'/exp OR trial))) AND ([controlled clinical trial]/lim OR [randomized controlled trial]/lim) AND [humans]/lim AND [abstracts]/lim AND [clinical study]/lim

**Web of science: 220**

https://www.webofscience.com/wos/alldb/summary/ecea556c-737e-4f1d-ad49-39871dcafa4a-016750084f/relevance/1

((Methotrexate) OR (MTX)) AND (rheumatoid arthritis) AND (subcutaneous OR parenteral OR injectable OR intramuscular) AND ((oral) OR (tablet)) AND (clinical trial)

Refined by: (NOT Database: Preprint Citation Index.) (Document Types: Article or Clinical Trial or Abstract or Editorial Material or Letter or Case Report or Dissertation Thesis)

**Cochrane: 279**

((Methotrexate) OR (MTX)) AND (rheumatoid arthritis) AND (subcutaneous OR parenteral OR injectable OR intramuscular) AND ((oral) OR (tablet)) AND (clinical trial)
