# Supplementary material for: Subcutaneous methotrexate compared with oral methotrexate in rheumatoid arthritis: a systematic review and meta-analysis
Source: Front Immunol. 2026 Jul 17;17:1816269. doi: 10.3389/fimmu.2026.1816269 (PMC13424238; doi:10.3389/fimmu.2026.1816269)
Supplement: Supplementary file 2 [file DataSheet2.docx]

Table S1. Characteristics of included studies

| **Study, year** | **country** | **Study design** | **No. of patients** | **Female (%)** | **Age (median (min, max) or mean (SD))** | | **Primary end point (week)** | **Disease duration (months)** | | **MTX dose (mg/week) min-max** | **MTX duration (week)** | **Outcomes (efficacy/AE/bioavailability)** |
| --- | --- | --- | --- | --- | --- | --- | --- | --- | --- | --- | --- | --- |
|  |  |  |  |  | OR MTX | SC MTX |  | OR MTX | SC MTX |  |  |  |
| L.Qiao, et al. 2025[1] | China | RCT | 137 | 82.5% | 52.3(9.67) | 49.4(9.64) | DAS28-ESR(12 week） | Median(Q1,Q3)  6.86(0.10,55.79) | Median(Q1,Q3)  2.95(0.40,16.32) | 15 mg/week | 12 weeks | Efficacy & AE |
| Braun.J, et al. 2008[2] | Germany | RCT | 375 | 75% | 59 (22–75) | 58 (20–75) | ACR20(24 week) | Median(range)  2.1(0-293) | Median(range)  2.5(0-535) | 15 mg/week | 24 weeks | Efficacy & AE |
| Michael H Schiff， et al. 2014[3] | USA | Randomized Cross-over trial | 49 | 63.3% | 61.4 (10.5) | | AUC_0–t_  C_max_ | 159.6（105） | 159.6（105） | 10~25mg/week | 8 weeks | Bioavailability & AE |
| Dhaon et al., 2018 [4] | India | RCT | 135 | 85.2% | 41(10) | 42 (11) | Low disease activity (LDA)(24 week) \| SDAI change from baseline (24 week) | Mean(sd)  67(75)  65(59) | Mean(sd)  68(54) | 15~22.5 mg/week | 24 weeks | Efficacy & AE |
| U. Pichlmeier et al. 2014[5] | Germany | Randomized Cross-over trial | 65 | 21.5% | 41.12 ( 12.06) | | AUC_0–t_  C_max_ | Not applicable | | 15mg | Single dose | Bioavailability & AE |
| S. J. CARMICHAEL, et.al.  2002[6] | Australia | Randomized Cross-over trial | 10 | 40% | 20-48 | | AUC_0–t_  C_max_  T_max_ | Not applicable | | 15mg | Single dose | Bioavailability |
| P. Seideman, et.al. 1993[7] | Sweden | Randomized Cross-over trial | 9 | unknown | 60(47-72) | | AUC  24h urinary excretion | unknown | | 15mg | Single dose with >=1-week intervals | Bioavailability |
| Y. Tanaka, et.al. 2023[8] | Japan | RCT | 102 | 80.4% | 54.1(12.20) | 57.8(10.84) | ACR20 (12 week) | Mean(sd)  0.91 (1.56) | Mean(sd)  1.33 (4.15) | 7.5~8mg | 12 weeks | Efficacy & AE |
| Islam MS , et al. 2013[9] | Bangladesh | RCT | 92 | 81.5% | 44.63(13.99) | 45.54(12.42) | ACR20(24 week)  ACR50 (24 week)  ACR70 (24 week) | Mean  49 | Mean  49.74 | unknown | 24 weeks (reported as six months) | Efficacy & AE |

Table S1 (continued). MTX treatment context, centre setting and assessment windows requested by reviewers

| **Study** | **Region/country** | **Centre setting** | **MTX route/dose** | **Background/concomitant therapy** | **Assessment window** | **Classification note** |
| --- | --- | --- | --- | --- | --- | --- |
| Qiao et al., 2025 | Asia; China | Multicentre phase III RCT; 20 tertiary hospitals in China; open-label, evaluator-blinded | SC MTX 15 mg/week vs oral MTX 15 mg/week for 12 weeks | DMARD-naive or DMARDs stopped >=4 weeks; stable NSAIDs and/or glucocorticoids (<=10 mg/day) allowed; folic acid 5 mg weekly 24 h after MTX | DAS28-ESR at 12 weeks; ACR20/50/70 at 4, 8, and 12 weeks; AEs through 12 weeks | MTX was not necessarily strict monotherapy because stable symptom-control therapy was allowed; no concomitant DMARDs during trial were described |
| Braun et al., 2008 | Europe; Germany | Multicentre phase IV RCT; 29 centres in Germany; randomized, double-blind, double-dummy | SC MTX 15 mg/week vs oral MTX 15 mg/week for 24 weeks; week-16 nonresponders followed protocol-defined switch/escalation | MTX-naive; biologics prohibited; other DMARDs discontinued before randomization and during study; stable NSAIDs and corticosteroids <=10 mg/day permitted; folic acid 5 mg after MTX | ACR and safety assessments at baseline, week 4, every 2 weeks to week 12, week 16, and week 24; main efficacy endpoint at 24 weeks | No concomitant DMARDs during the randomized comparison; stable steroids/NSAIDs permitted |
| Schiff et al., 2014 | Americas; USA | Multicentre phase II randomized-sequence crossover study; four clinical sites in the USA; open-label | Oral MTX vs SC MTX auto-injector into abdomen and thigh; 10, 15, 20, or 25 mg/week according to current regimen | RA patients treated with MTX for >=3 months; concomitant medications stable for >=3 months; additional DMARDs that could interfere with PK outcomes excluded; NSAIDs not permitted within +/-12 h of MTX administration | PK samples predose to 24 h after each route; safety across the 8-week crossover period | Bioavailability/safety study; not an efficacy RCT |
| Dhaon et al., 2018 | Asia; India | Department-based 24-week randomized trial at KGMU, India | Parenteral/IM MTX 15 or 22.5 mg/week vs oral MTX 15 or 22.5 mg/week; dose increased at week 6 if disease activity persisted | Patients were on 7.5 mg oral MTX for >=4 weeks at entry; no other DMARDs except MTX and hydroxychloroquine in prior 3 months; all received HCQ 400 mg, folic acid, methylprednisolone for first 4 weeks, and etoricoxib as needed | Clinical evaluations every 6 weeks for 24 weeks; LDA/SDAI and extracted ACR20/50/70 at 24 weeks; AEs through 24 weeks | Combination background therapy with HCQ and short-course steroid was used; parenteral arm was intramuscular rather than SC |
| Pichlmeier et al., 2014 | Europe; Germany | Single-centre, open-label, randomized 2-period crossover PK study in Mannheim, Germany | Single-dose SC MTX pen vs oral MTX tablets at 7.5, 15, 22.5, or 30 mg | Healthy subjects; folinic acid and citrate co-medication were administered for safety | PK sampling to 48 h after each route; AEs after single-dose exposure | Bioavailability/safety study; no RA DMARD background |
| Carmichael et al., 2002 | Oceania; Australia | Randomized crossover PK interaction study approved at St Vincent's Hospital | 15 mg oral MTX, 15 mg IV MTX, 200 mg HCQ, and oral MTX plus HCQ dosing occasions | Healthy volunteers; designed to assess MTX-HCQ pharmacokinetic interaction rather than RA clinical efficacy | PK sampling to 96 h after dosing | Bioavailability study; not an MTX monotherapy RA efficacy trial |
| Seideman et al., 1993 | Europe; Sweden | Randomized crossover PK study at Karolinska/Danderyd Hospital, Sweden | 15 mg MTX given IV, IM, and orally at >=1-week intervals | Nine RA patients with current inflammatory activity about to start MTX; no other drugs allowed from 3 days before the study until completion | Plasma sampling to 168 h and 24-h urinary excretion after dosing | Bioavailability study; no concomitant RA drugs during the PK assessment |
| Tanaka et al., 2023 | Asia; Japan | Multicentre trial at 37 sites in Japan; Part 1 randomized, double-blind, active-controlled, double-dummy | Part 1: SC MTX 7.5 mg/week vs oral MTX 8 mg/week for 12 weeks; Part 2: SC MTX 7.5-15 mg/week extension | MTX-naive active RA patients; folic acid was given concomitantly (5 mg in Part 1; 5 or 10 mg in Part 2) | ACR20/50/70 and DAS28-ESR at 4, 8, and 12 weeks in Part 1; extension assessments through week 64 | Main meta-analysis used the 12-week randomized comparison; no concomitant DMARD background was described for Part 1 |
| Islam et al., 2013 | Asia; Bangladesh | Prospective trial conducted in the rheumatology clinic of Bangabandhu Sheikh Mujib Medical University | Injectable/subcutaneous MTX vs oral MTX; dose not reported in the available local abstract/full-text file | Concomitant DMARD or background therapy information was not reported in the available local abstract/full-text file | ACR20/50/70 and AEs at 24 weeks | Treatment-background details remain unclear from the available report |

Notes: AE, adverse event; DMARD, disease-modifying antirheumatic drug; HCQ, hydroxychloroquine; IM, intramuscular; IV, intravenous; MTX, methotrexate; PK, pharmacokinetic; SC, subcutaneous. Folic acid and stable symptom-control therapy are listed separately from concomitant DMARD therapy.

1. Qiao L, Hu J, Ou D, Liu M, Shi X, Li X, Wu R, Zhang L, Liu Y, Xiao C: **Comparison of the efficacy and safety of methotrexate injection and methotrexate tablets in active active rheumatoid arthritis**. *Rheumatology* 2025:keaf054.

2. Braun J, Kästner P, Flaxenberg P, Währisch J, Hanke P, Demary W, Von Hinüber U, Rockwitz K, Heitz W, Pichlmeier U: **Comparison of the clinical efficacy and safety of subcutaneous versus oral administration of methotrexate in patients with active rheumatoid arthritis: results of a six‐month, multicenter, randomized, double‐blind, controlled, phase IV trial**. *Arthritis & Rheumatism* 2008, **58**(1):73-81.

3. Schiff MH, Jaffe JS, Freundlich B: **Head-to-head, randomised, crossover study of oral versus subcutaneous methotrexate in patients with rheumatoid arthritis: drug-exposure limitations of oral methotrexate at doses≥ 15 mg may be overcome with subcutaneous administration**. *Annals of the rheumatic diseases* 2014, **73**(8):1549-1551.

4. Dhaon P, Das SK, Srivastava R, Agarwal G, Asthana A: **Oral methotrexate in split dose weekly versus oral or parenteral methotrexate once weekly in rheumatoid arthritis: a short‐term study**. *International Journal of Rheumatic Diseases* 2018, **21**(5):1010-1017.

5. Pichlmeier U, Heuer K-U: **Subcutaneous administration of methotrexate with a prefilled autoinjector pen results in a higher relative bioavailability compared with oral administration of methotrexate**. *Clin Exp Rheumatol* 2014, **32**(4):563-571.

6. Carmichael SJ, Beal J, Day RO, Tett SE: **Combination therapy with methotrexate and hydroxychloroquine for rheumatoid arthritis increases exposure to methotrexate**. *The Journal of rheumatology* 2002, **29**(10):2077-2083.

7. Seideman P, Beck O, Eksborg S, Wennberg M: **The pharmacokinetics of methotrexate and its 7‐hydroxy metabolite in patients with rheumatoid arthritis**. *British journal of clinical pharmacology* 1993, **35**(4):409-412.

8. Tanaka Y, Okuda K, Takeuchi Y, Katayama K, Haji Y, Yamanishi Y, Tribanek M, Guimbal-Schmolck C, Takeuchi T: **Efficacy and tolerability of subcutaneously administered methotrexate including dose escalation in long-term treatment of rheumatoid arthritis in a Japanese population**. *Modern rheumatology* 2023, **33**(4):680-689.

9. Islam M, Haq S, Islam M, Azad A, Islam M, Barua R, Hasan M, Mahmood M, Safiuddin M, Rahman M: **Comparative efficacy of subcutaneous versus oral methotrexate in active rheumatoid arthritis**. *Mymensingh Medical Journal: MMJ* 2013, **22**(3):483-488.

Table S2. Fixed-effect sensitivity analyses for outcomes with I2 <50%

| **Outcome** | **No. studies** | **I2 (%)** | **Primary random-effects result** | **Fixed-effect sensitivity result** | **Interpretation** |
| --- | --- | --- | --- | --- | --- |
| ACR20 | 5 | 0.0 | RR=1.15 (1.05, 1.25); P=0.0013 | RR=1.15 (1.05, 1.25); P=0.0025 | Inference consistent; statistically significant in both models. |
| ACR50 | 5 | 0.0 | RR=1.14 (1.01, 1.29); P=0.0389 | RR=1.12 (0.99, 1.28); P=0.0717 | Fixed-effect result attenuated; primary random-effects finding should be interpreted cautiously. |
| ACR70 (final dataset incl. Braun 2008) | 5 | 41.9 | RR=1.29 (0.81, 2.05); P=0.2800 | RR=1.26 (0.999, 1.599); P=0.0508 | Fixed-effect result was borderline (P=0.0508), but the primary random-effects result remained non-significant; retain the primary non-significant inference. |
| DAS28-ESR | 2 | 33.7 | MD=-0.12 (-0.34, 0.11); P=0.2994 | MD=-0.17 (-0.22, -0.12); P=<0.001 | Fixed-effect result was significant but primary random-effects result was not; retain primary random-effects inference. |
| TEAE | 2 | 0.0 | RR=0.77 (0.58, 1.04); P=0.0842 | RR=0.77 (0.58, 1.03); P=0.0815 | Inference consistent; not statistically significant in either model. |
| any AE | 3 | 47.7 | RR=0.93 (0.75, 1.17); P=0.5555 | RR=0.99 (0.87, 1.12); P=0.8293 | Inference consistent; not statistically significant in either model. |
| Gastrointestinal system disorders | 4 | 0.0 | RR=0.58 (0.40, 0.83); P=0.0033 | RR=0.57 (0.40, 0.83); P=0.0028 | Inference consistent; statistically significant in both models. |
| Diarrhea | 4 | 0.0 | RR=0.42 (0.21, 0.84); P=0.0141 | RR=0.40 (0.20, 0.78); P=0.0078 | Inference consistent; statistically significant in both models. |
| Nausea | 6 | 41.6 | RR=0.75 (0.45, 1.27); P=0.2826 | RR=0.83 (0.61, 1.12); P=0.2298 | Inference consistent; not statistically significant in either model. |
| Vomiting | 3 | 22.1 | RR=0.62 (0.27, 1.42); P=0.2588 | RR=0.60 (0.32, 1.14); P=0.1189 | Inference consistent; not statistically significant in either model. |
| Abdominal pain | 2 | 0.0 | RR=0.80 (0.44, 1.47); P=0.4740 | RR=0.79 (0.44, 1.45); P=0.4548 | Inference consistent; not statistically significant in either model. |
| Abdominal pain upper | 2 | 0.0 | RR=0.14 (0.02, 1.14); P=0.0664 | RR=0.14 (0.02, 1.14); P=0.0664 | Inference consistent; not statistically significant in either model. |
| Dyspepsia | 2 | 46.9 | RR=0.78 (0.41, 1.48); P=0.4436 | RR=0.78 (0.50, 1.22); P=0.2771 | Inference consistent; not statistically significant in either model. |
| Stomatitis | 2 | 0.0 | RR=0.87 (0.36, 2.11); P=0.7645 | RR=0.87 (0.36, 2.11); P=0.7629 | Inference consistent; not statistically significant in either model. |
| Musculoskeletal and connective tissue disorders | 2 | 0.0 | RR=1.08 (0.39, 2.95); P=0.8834 | RR=1.12 (0.41, 3.01); P=0.8292 | Inference consistent; not statistically significant in either model. |

Notes: AE, adverse event; CI, confidence interval; DAS28-ESR, 28-joint Disease Activity Score for erythrocyte sedimentation rate; GI, gastrointestinal; I2, inconsistency statistic; MD, mean difference; RR, relative risk; TEAE, treatment-emergent adverse event. Fixed-effect analyses were conducted as sensitivity analyses for outcomes with I2 <50%; the primary model remained the random-effects model because clinical heterogeneity was expected across studies.

Table S3. Exploratory subgroup analyses by assessment time point and geographic region

| **Outcome** | **Subgroup factor** | **Subgroup** | **No. studies** | **RR (95% CI)** | **I2 (%)** | **Studies** |
| --- | --- | --- | --- | --- | --- | --- |
| ACR20 | Assessment time point | 12 weeks | 2 | 1.28 (1.06-1.56) | 0.0 | Qiao 2025; Tanaka 2023 |
| ACR20 | Assessment time point | 24 weeks | 3 | 1.12 (1.02-1.22) | 0.0 | Braun 2008; Dhaon 2018; Islam 2013 |
| ACR20 | Geographic region | Asia | 4 | 1.17 (1.05-1.32) | 0.0 | Qiao 2025; Dhaon 2018; Tanaka 2023; Islam 2013 |
| ACR20 | Geographic region | Europe | 1 | 1.12 (0.99-1.26) | 0.0 | Braun 2008 |
| ACR50 | Assessment time point | 12 weeks | 2 | 1.28 (0.90-1.81) | 0.0 | Qiao 2025; Tanaka 2023 |
| ACR50 | Assessment time point | 24 weeks | 3 | 1.12 (0.99-1.27) | 0.0 | Braun 2008; Dhaon 2018; Islam 2013 |
| ACR50 | Geographic region | Asia | 4 | 1.23 (1.04-1.46) | 0.0 | Qiao 2025; Dhaon 2018; Tanaka 2023; Islam 2013 |
| ACR50 | Geographic region | Europe | 1 | 1.06 (0.90-1.25) | 0.0 | Braun 2008 |
| ACR70 | Assessment time point | 12 weeks | 2 | 1.41 (0.27-7.45) | 83.5 | Qiao 2025; Tanaka 2023 |
| ACR70 | Assessment time point | 24 weeks | 3 | 1.22 (0.95-1.57) | 0.0 | Braun 2008; Dhaon 2018; Islam 2013 |
| ACR70 | Geographic region | Asia | 4 | 1.29 (0.60-2.76) | 55.6 | Qiao 2025; Dhaon 2018; Tanaka 2023; Islam 2013 |
| ACR70 | Geographic region | Europe | 1 | 1.24 (0.95-1.61) | 0.0 | Braun 2008 |

Notes: Exploratory post hoc analyses used inverse-variance pooling of log risk ratios with a random-effects variance component. Strata with one study are descriptive only. Americas and Oceania contributed bioavailability/PK studies but no eligible ACR20/50/70 efficacy RCTs; therefore regional/ethnic comparisons should be interpreted cautiously.

Table S4. Feasibility of 4-, 8-, 12-, and 24-week efficacy subgroup analyses

| **Study** | **Route comparison** | **4-week data** | **8-week data** | **12-week data** | **24-week data** | **Feasibility decision** |
| --- | --- | --- | --- | --- | --- | --- |
| Qiao et al., 2025 | SC MTX 15 mg/week vs oral MTX 15 mg/week | Reported descriptively for ACR20/50 and ACR70 in text/Figure 3; exact denominator-consistent arm-level counts were not fully extractable from the local article text. | Reported in Figure 3, but exact arm-level ACR20/50/70 event counts were not available in the local extractable text. | Extractable and used in the 12-week subgroup/meta-analysis. | Not applicable; randomized follow-up was 12 weeks. | Included in 12-week pooled subgroup; 4/8-week results described qualitatively only. |
| Tanaka et al., 2023 | SC MTX 7.5 mg/week vs oral MTX 8 mg/week | Exact counts available: ACR20 19/52 vs 16/49; ACR50 4/52 vs 4/49; ACR70 1/52 vs 2/49. | Exact counts available: ACR20 24/52 vs 24/49; ACR50 10/52 vs 12/49; ACR70 3/52 vs 4/49. | Exact counts available and used in the 12-week subgroup/meta-analysis. | Not applicable for randomized comparison; later extension was single-arm/open-label. | Only study with complete exact 4/8-week ACR counts; insufficient alone for formal subgroup meta-analysis. |
| Braun et al., 2008 | SC MTX 15 mg/week vs oral MTX 15 mg/week | Trial visits included week 4, but exact arm-level ACR20/50/70 counts for week 4 were not reported in an extractable table. | Trial visits occurred through week 12, but exact arm-level ACR20/50/70 counts for week 8 were not reported in an extractable table. | Not used as primary extractable endpoint; the trial's main endpoint was week 24. | Extractable and used in the 24-week subgroup/meta-analysis. | Included in 24-week pooled subgroup; 4/8-week pooling would require unpublished data or figure digitization. |
| Dhaon et al., 2018 | Parenteral/IM MTX 15-22.5 mg/week vs oral MTX 15-22.5 mg/week | Not aligned with reported efficacy schedule. | Not aligned with reported efficacy schedule. | Not extractable for ACR20/50/70 from the local report. | Extractable and used in the 24-week subgroup/meta-analysis. | Included in 24-week pooled subgroup and route sensitivity analysis. |
| Islam et al., 2013 | Injectable/subcutaneous MTX vs oral MTX; dose not reported in local file | Not reported in the available local abstract/full-text file. | Not reported in the available local abstract/full-text file. | Not reported in the available local abstract/full-text file. | Extractable and used in the 24-week subgroup/meta-analysis. | Included in 24-week pooled subgroup; no early time-point analysis possible. |

Notes: ACR, American College of Rheumatology response; IM, intramuscular; MTX, methotrexate; SC, subcutaneous. Formal 4- and 8-week pooled analyses were not conducted because exact arm-level early ACR event counts were not consistently extractable across studies.

Table S5. Dose-specific evidence and route-focused sensitivity analyses

| **Issue** | **Analysis** | **Studies/data** | **Estimate/observation** | **Interpretation** |
| --- | --- | --- | --- | --- |
| Low-dose clinical evidence (<15 mg/week) | Single low-dose randomized efficacy comparison | Tanaka 2023; SC MTX 7.5 mg/week vs oral MTX 8 mg/week for 12 weeks | ACR20 RR=1.17 (0.82-1.67); ACR50 RR=1.10 (0.57-2.14); ACR70 RR=0.59 (0.21-1.68) | Only one clinical RCT directly informs <15 mg/week; no formal low-dose subgroup meta-analysis is possible. |
| Dose category around 15 mg/week | Descriptive efficacy estimate for 15 mg/week studies | Qiao 2025 and Braun 2008; both used 15 mg/week, but endpoint timing differed (12 vs 24 weeks) | ACR20 RR=1.19 (1.00-1.41); ACR50 RR=1.11 (0.92-1.34); ACR70 RR=1.82 (0.73-4.57) | Suggestive direction favouring SC/parenteral MTX for ACR20, but mixed timing and small k preclude dose-response inference. |
| Variable or higher dose category | Single variable-dose parenteral comparison | Dhaon 2018; IM/parenteral MTX 15 or 22.5 mg/week vs oral MTX 15 or 22.5 mg/week | ACR20 RR=0.97 (0.71-1.32); ACR50 RR=1.00 (0.56-1.79); ACR70 RR=1.00 (0.38-2.62) | This study is informative for parenteral/IM MTX but not a strict SC-vs-oral sensitivity stratum. |
| Route sensitivity for efficacy | Main ACR analyses after excluding the IM/parenteral Dhaon 2018 trial | Qiao 2025, Braun 2008, Tanaka 2023, Islam 2013 | ACR20 RR=1.16 (1.06-1.27), I2=0.0%; ACR50 RR=1.14 (1.01-1.29), I2=0.0%; ACR70 RR=1.35 (0.76-2.41), I2=54.8% | The significant ACR20/ACR50 findings are robust to excluding the IM/parenteral efficacy study; ACR70 remains non-significant. |
| Route sensitivity for bioavailability | AUC restricted to SC-vs-oral studies; Cmax already came from SC-vs-oral studies | AUC: Schiff 2014 and Pichlmeier 2014 only; Cmax: Schiff 2014 and Pichlmeier 2014 | AUC MD=264.50 (-218.01 to 747.00), I2=95.9%; Cmax MD=36.46 (-49.33 to 122.24), I2=81.9% | SC-only bioavailability estimates remain imprecise; the data do not establish a pooled low-dose PK advantage below 15 mg/week. |

Notes: IM, intramuscular; MTX, methotrexate; PK, pharmacokinetic; SC, subcutaneous. Estimates are exploratory and were calculated to clarify dose and route heterogeneity; one-study strata are descriptive only.

Table S6. Clinically important MTX toxicities not pooled quantitatively

| **Toxicity domain** | **Examples of available reporting** | **Why not pooled** | **How handled** |
| --- | --- | --- | --- |
| Serious or severe adverse events | Qiao reported SAE, drug-related SAE, severe TEAE and drug-related severe TEAE; Braun reported serious adverse events; Pichlmeier reported no serious events after single-dose exposure; Tanaka reported severe AEs/SAEs in Part 1. | Definitions, populations and exposure windows differed substantially, and some studies were single-dose pharmacokinetic studies rather than RA efficacy trials. | Summarized descriptively; no route-specific serious toxicity conclusion was inferred. |
| Hepatic injury or liver-function abnormality | Qiao reported ALT/AST increases and hepatic-function abnormality; Tanaka reported ALT/liver-function test increases; Dhaon reported withdrawal due to transaminitis; Pichlmeier described reversible liver-enzyme increases. | Reports mixed laboratory abnormalities, hepatobiliary system-organ-class events and withdrawal reasons; denominator and follow-up windows were not harmonized across trials. | Listed as non-pooled clinically important MTX toxicity; interpreted as insufficient for comparative meta-analysis. |
| Renal impairment or renal/urinary disorder | Pichlmeier reported sparse renal/urinary events in dose strata; several trials monitored or excluded renal impairment but did not report extractable arm-level renal toxicity events. | Events were rare, inconsistently defined and often not available as arm-level MTX-route comparisons. | No pooled estimate; acknowledged as an important toxicity requiring better prospective reporting. |
| Myelosuppression or bone-marrow/hematologic toxicity | Qiao reported white blood cell and lymphocyte count decreases and anaemia; Dhaon reported withdrawals due to hematological disorder; Braun excluded impaired hematopoiesis at baseline. | Available data used different labels and severity thresholds, and did not consistently distinguish mild laboratory shifts from clinically important myelosuppression. | No pooled estimate; summarized descriptively as non-comparable hematologic safety reporting. |
| Pulmonary toxicity or interstitial lung disease | Dhaon reported one withdrawal due to interstitial lung disease; Tanaka reported respiratory/thoracic/mediastinal system-organ-class events; Braun excluded clinically relevant pulmonary disease. | Pulmonary toxicity was rare and not consistently reported as route-specific, arm-level, clinically adjudicated MTX toxicity. | No pooled estimate; noted as insufficient evidence rather than evidence of no difference. |

Notes: AE, adverse event; ALT, alanine aminotransferase; AST, aspartate aminotransferase; MTX, methotrexate; SAE, serious adverse event; TEAE, treatment-emergent adverse event. These rows explain why clinically important but sparsely and inconsistently reported MTX toxicities were summarized narratively rather than pooled.

Table S7. Integrated results of effectiveness and safety

| Outcomes | Summary of findings | | Risk of bias | inconsistency | indirectness | imprecision | Publication bias | Overall certainty of evidence |
| --- | --- | --- | --- | --- | --- | --- | --- | --- |
|  | No. studies | RR (95%CI)/  MD (95%CI) |  |  |  |  |  |  |
| ACR20 | 5 | 1.15 (1.05-1.25) | Low | low | Low | Low | Low | High |
| ACR50 | 5 | 1.14 (1.01-1.29) | low | low | low | Low | low | moderate |
| ACR70 | 5 | 1.29 (0.81-2.05) | low | moderate | low | high | low | low |
| DAS28-ESR | 2 | -0.12 (-0.34-0.11) | low | moderate | low | moderate | low | Moderate |
| AUC | 4 | 122.62 (-237.28-482.52) | low | high | low | high | low | Low |
| Cmax | 2 | 36.46 (-49.33-122.24) | low | high | low | high | low | low |
| Any AE | 3 | 0.93 (0.75-1.17) | low | moderate | low | moderate | low | Low |
| TEAE | 2 | 0.77 (0.58-1.04) | low | low | low | moderate | low | moderate |
| Gastrointestinal（GI） | 4 | 0.58 (0.4-0.83) | low | low | low | low | low | high |
| Nausea | 6 | 0.75 (0.45-1.27) | low | moderate | low | high | low | low |
| Vomiting | 3 | 0.62 (0.27-1.42) | low | moderate | low | high | low | low |
| Abdominal pain upper | 2 | 0.14 (0.02-1.14) | low | low | low | Moderate | low | moderate |
| Diarrhea | 4 | 0.42 (0.21-0.84) | Low | low | low | low | Low | high |
| Abdominal pain | 2 | 0.8 (0.44-1.47) | low | low | low | high | low | low |
| Dyspepsia | 2 | 0.78 (0.41-1.48) | low | high | low | high | low | low |
| Stomatitis | 2 | 0.87 (0.36-2.11) | low | low | low | high | low | low |
| Musculoskeletal and connective tissue disorders | 2 | 1.08 (0.39-2.95) | low | low | low | high | low | low |

Table S8. Results of Egger’s test

| Outcomes | Number of studies | Egger’s test (t, p-value) |
| --- | --- | --- |
| ACR20 | 5 | t=0.0858, p=0.9370 |
| ACR50 | 5 | t = 0.3188, p = 0.7708 |
| ACR70 | 5 | t = 0.0248, p = 0.9818 |
| DAS28 | 2 | NA |
| AUC | 4 | t = 0.6033, p = 0.6076 |
| C_max_ | 2 | NA |
| TEAE | 2 | NA |
| GI | 4 | t = -1.1170, p = 0.3802 |
| Nausea | 6 | t = -0.6137, p = 0.5726 |
| Vomiting | 3 | t = 0.3438, p = 0.7892 |
| Abdominal pain upper | 2 | NA |
| Diarrhea | 4 | t = -1.7780, p = 0.2174 |
| Abdominal pain | 2 | NA |
| Any AE | 3 | t = -1.8479, p = 0.3158 |
| Dyspepsia | 2 | NA |
| Stomatitis | 2 | NA |
| Musculoskeletal and connective | 2 | NA |
