# Supplementary material for: Subcutaneous methotrexate compared with oral methotrexate in rheumatoid arthritis: a systematic review and meta-analysis
Source: Front Immunol. 2026 Jul 17;17:1816269. doi: 10.3389/fimmu.2026.1816269 (PMC13424238; doi:10.3389/fimmu.2026.1816269)
Supplement: Supplementary file 3 [file DataSheet3.docx]

**A. AUC0-t**
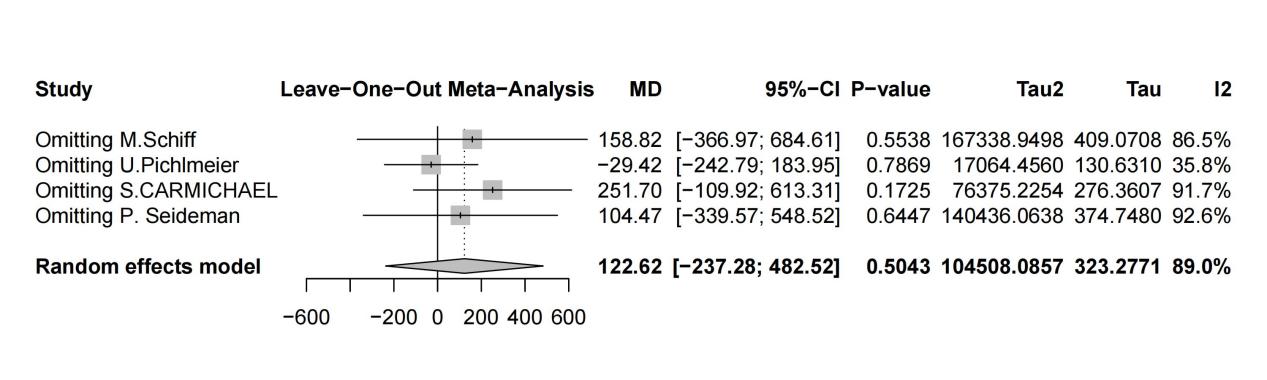
 **B. C_max_**
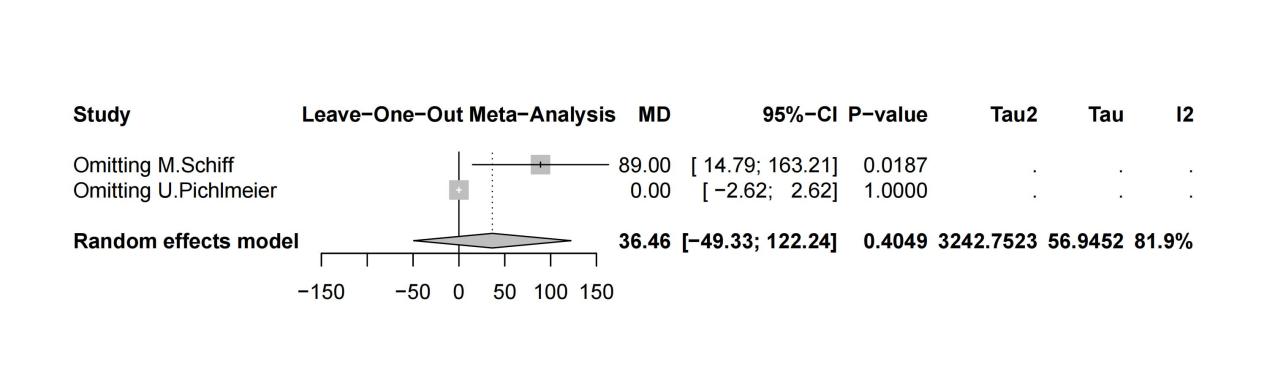


Figure S1. Leave-one-out sensitivity analyses for bioavailability outcomes: (A) AUC0-t; (B) C_max_. AUC0-t, area under the concentration-time curve from time zero to the last measurable concentration; C_max_, maximum plasma concentration.

| A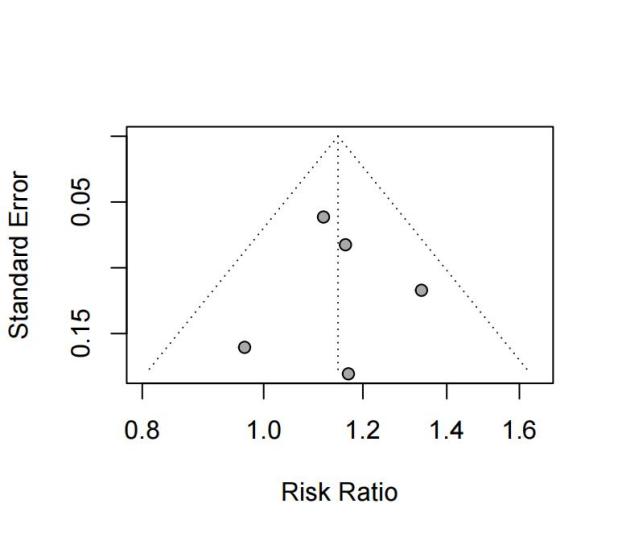 | B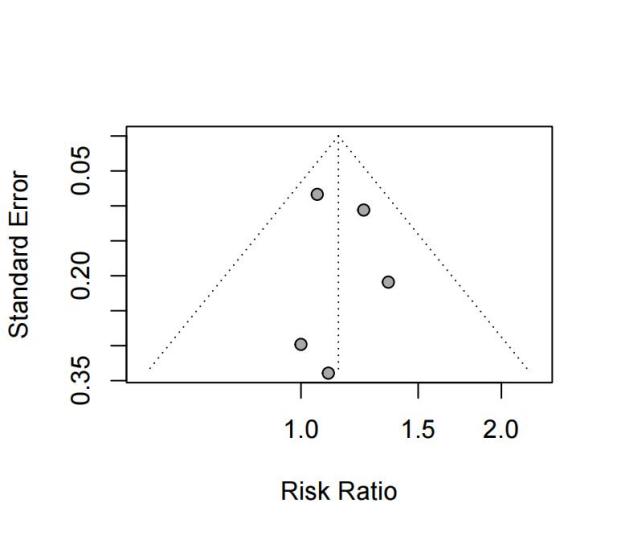 |
| --- | --- |
| C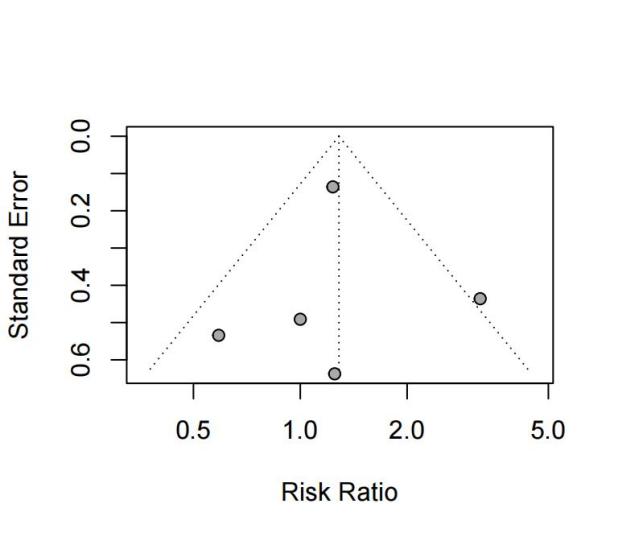 | D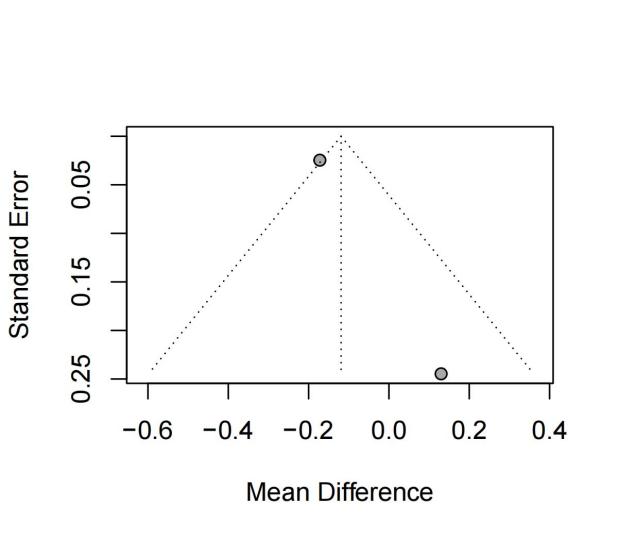 |

Figure S2. Funnel plots for publication bias assessment of efficacy outcomes: (A) ACR20 response; (B) ACR50 response; (C) ACR70 response; (D) DAS28-ESR.

| A  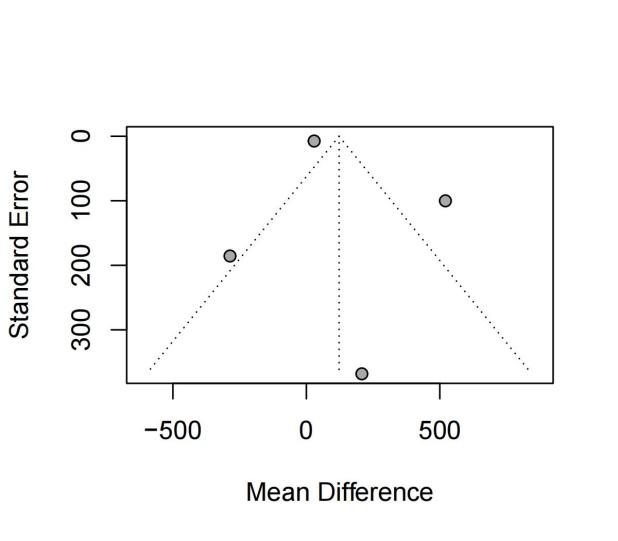 | B  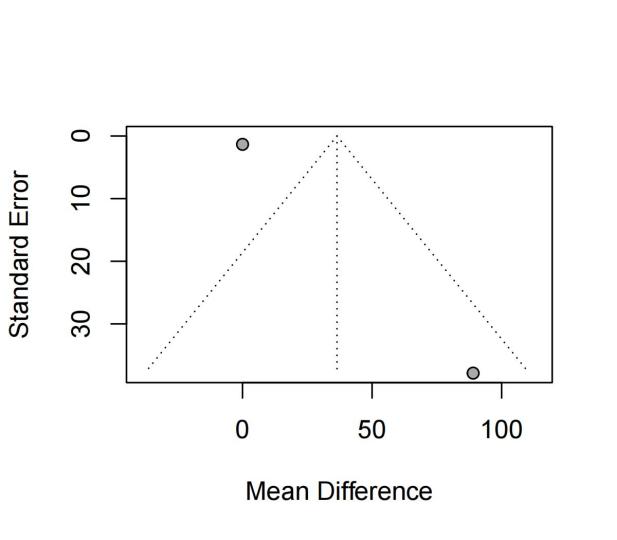 |
| --- | --- |

Figure S3. Funnel plots for publication bias assessment of bioavailability outcomes: (A) AUC0-t; (B) C_max_. AUC0-t, area under the concentration-time curve from time zero to the last measurable concentration; C_max_, maximum plasma concentration.

| A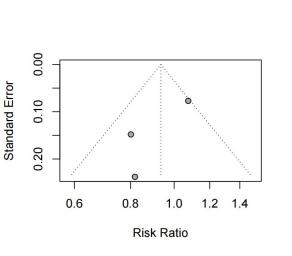 | B  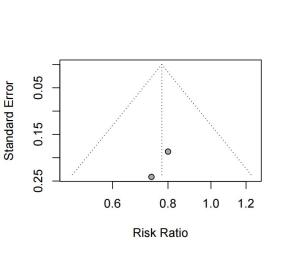 | C  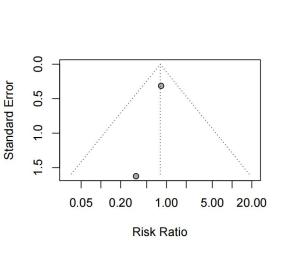 | D  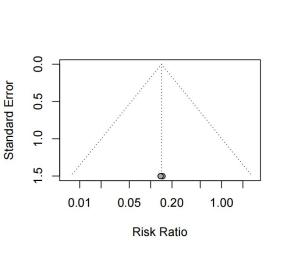 |
| --- | --- | --- | --- |
| E  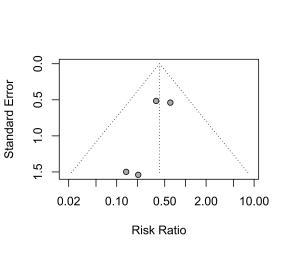 | F  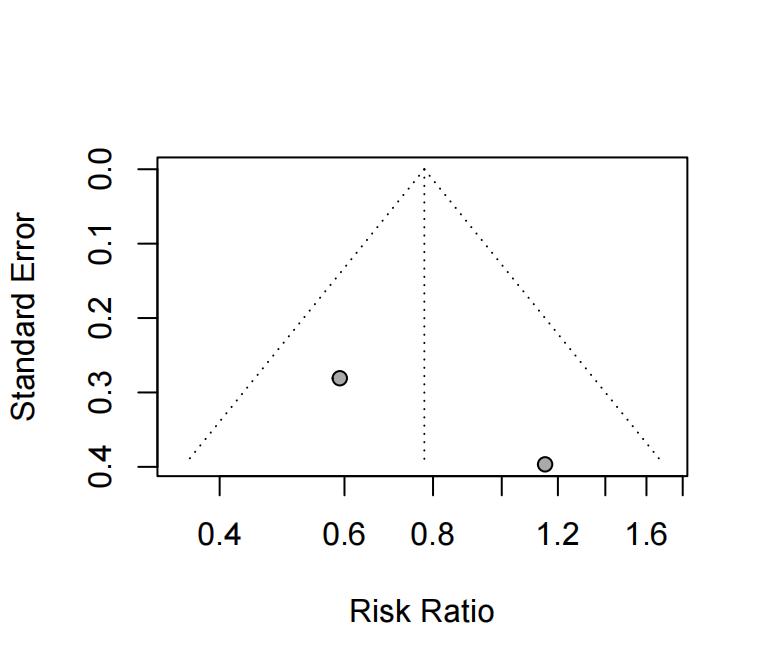 | G  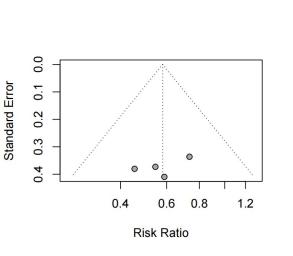 | H  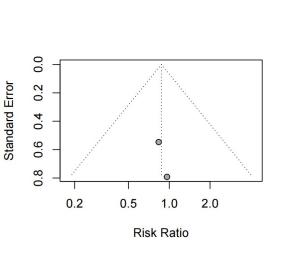 |
| I  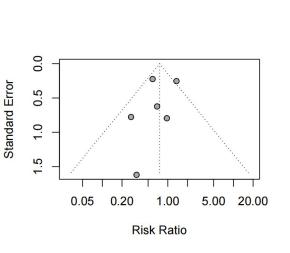 | J  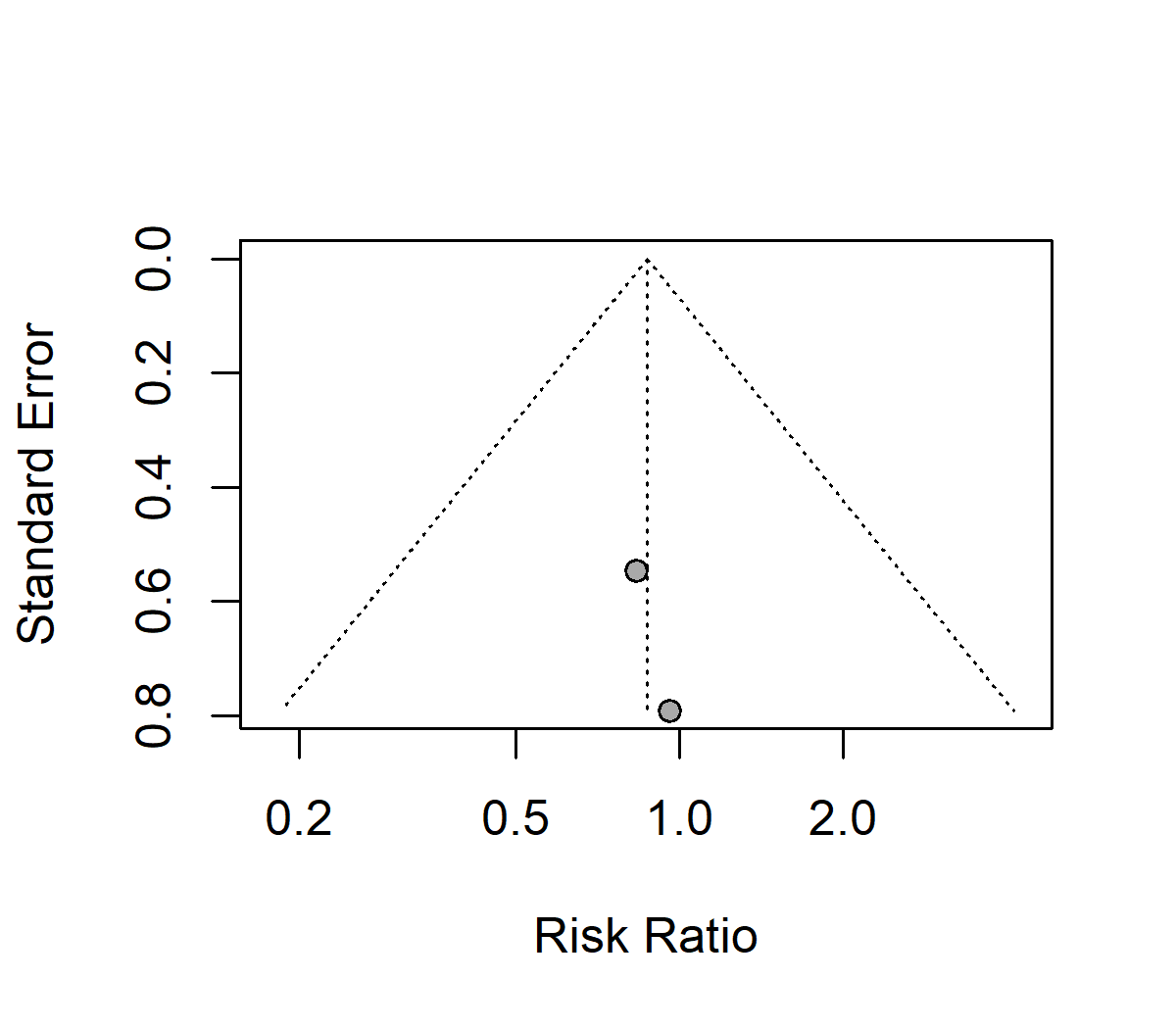 | K  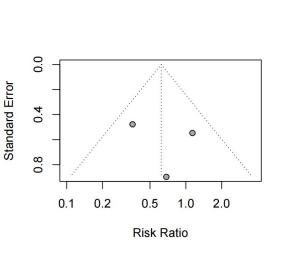 |  |

Figure S4. Funnel plots for publication bias assessment of safety outcomes: (A) any adverse event (AE); (B) treatment-emergent adverse events (TEAEs); (C) abdominal pain; (D) upper abdominal pain; (E) diarrhea; (F) dyspepsia; (G) gastrointestinal (GI) adverse events; (H) musculoskeletal and connective tissue disorders; (I) nausea; (J) stomatitis; (K) vomiting.

**A. ACR20 response**
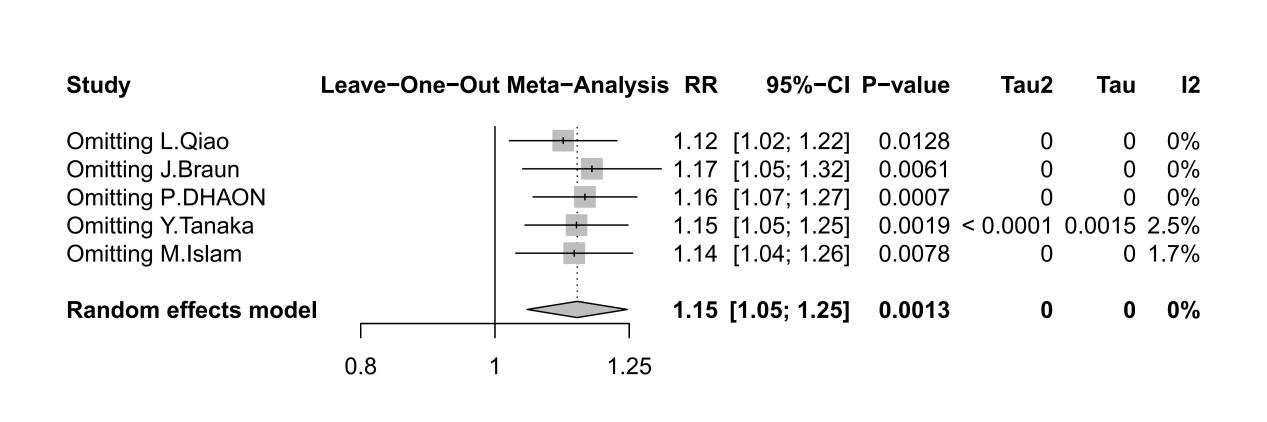


**B. ACR50 response**
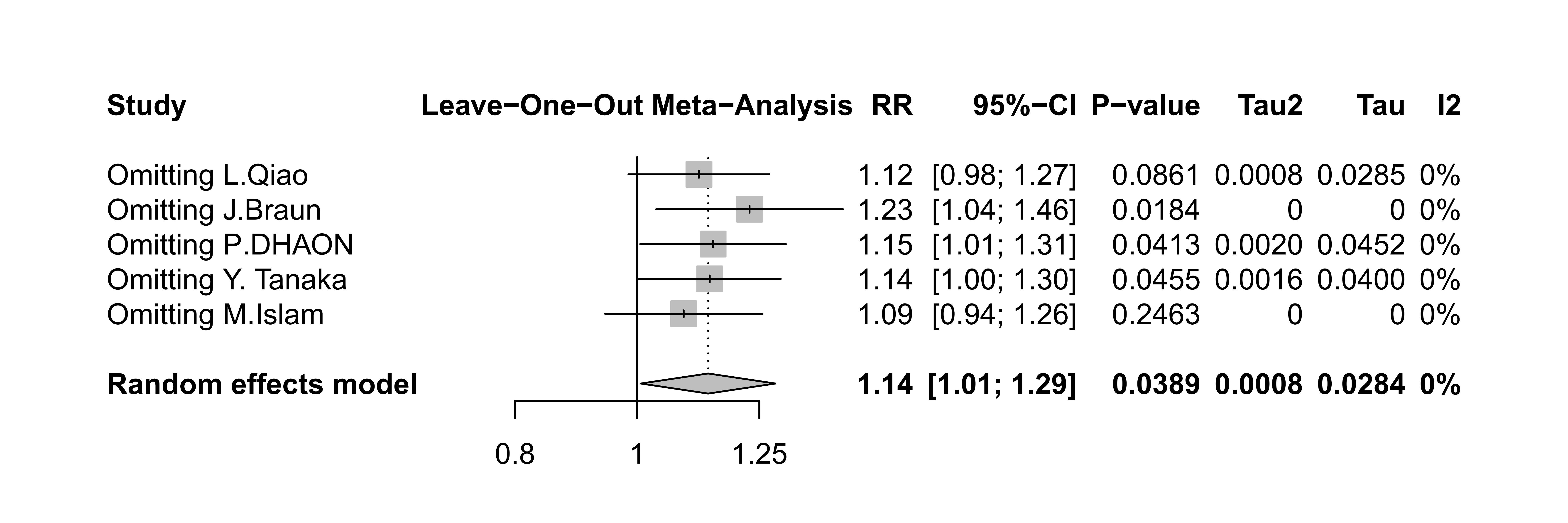


**C. ACR70 response**
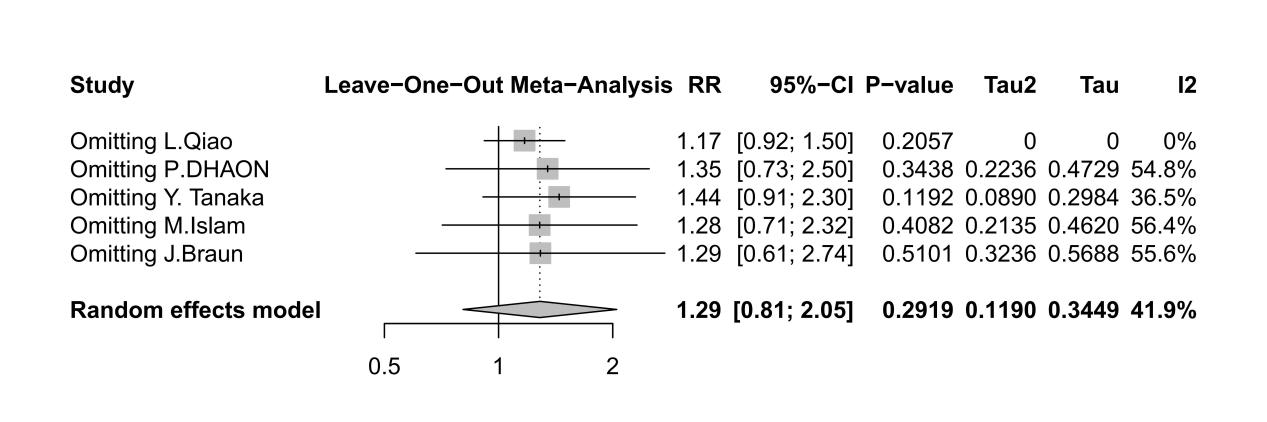


Figure S5. Leave-one-out sensitivity analyses for clinical response outcomes: (A) ACR20 response; (B) ACR50 response; (C) ACR70 response.


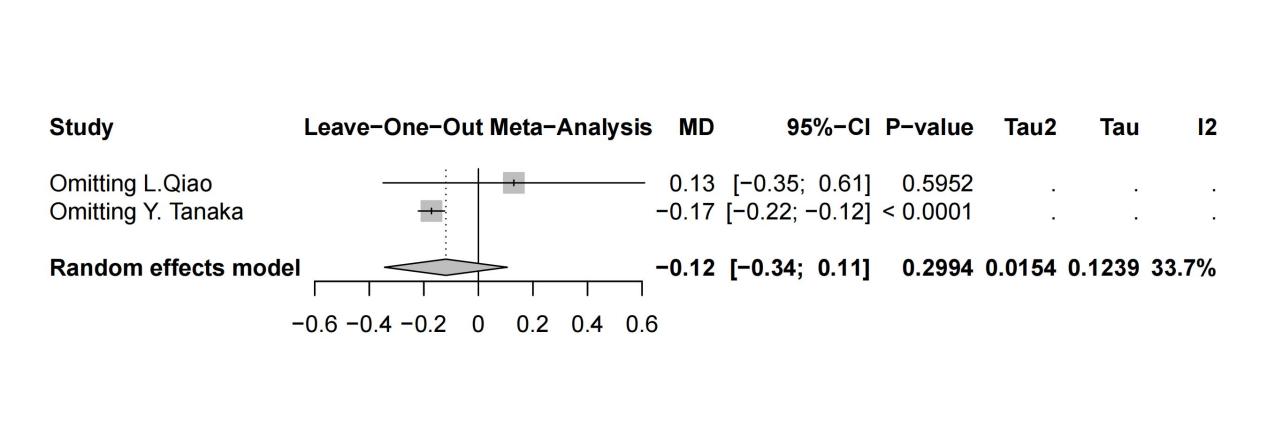


Figure S6. Leave-one-out sensitivity analysis for change in DAS28-ESR. DAS28-ESR, Disease Activity Score in 28 joints based on erythrocyte sedimentation rate.

**A. Any adverse event (AE)

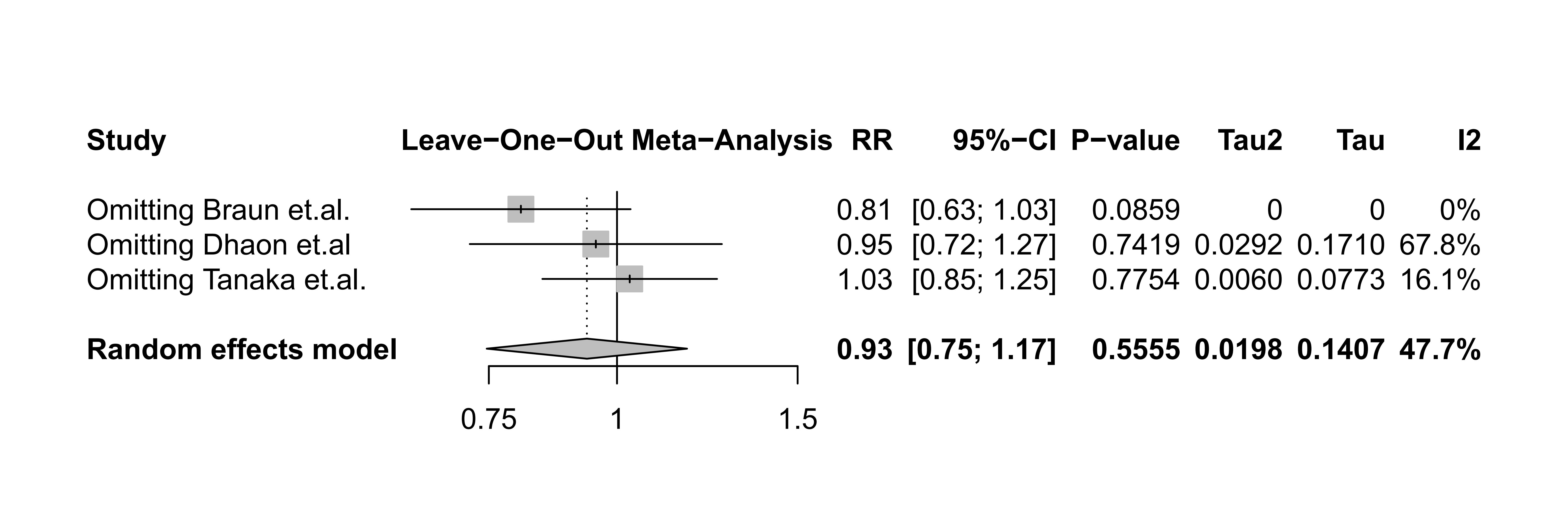
**

**B. Treatment-emergent adverse events (TEAEs)

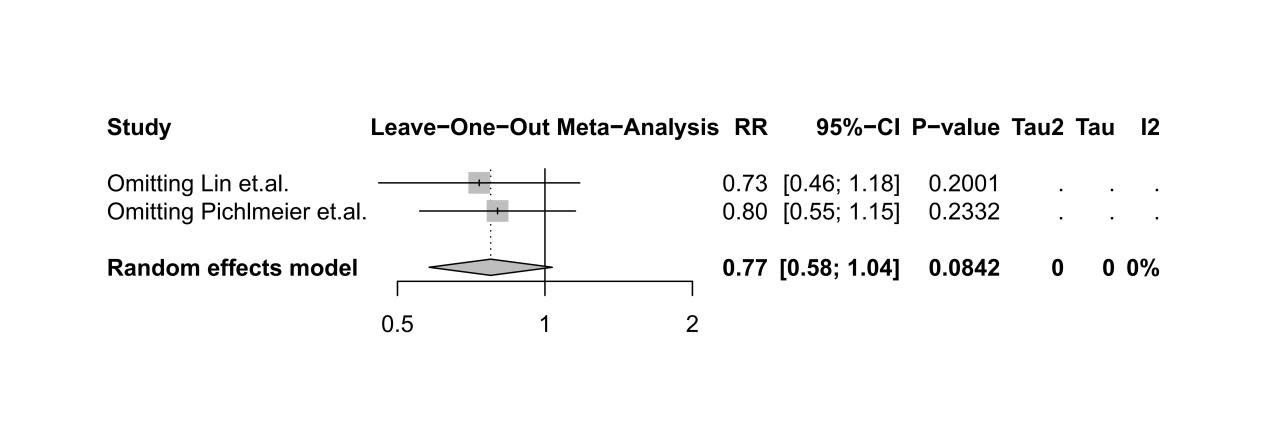
**

**C. Abdominal pain

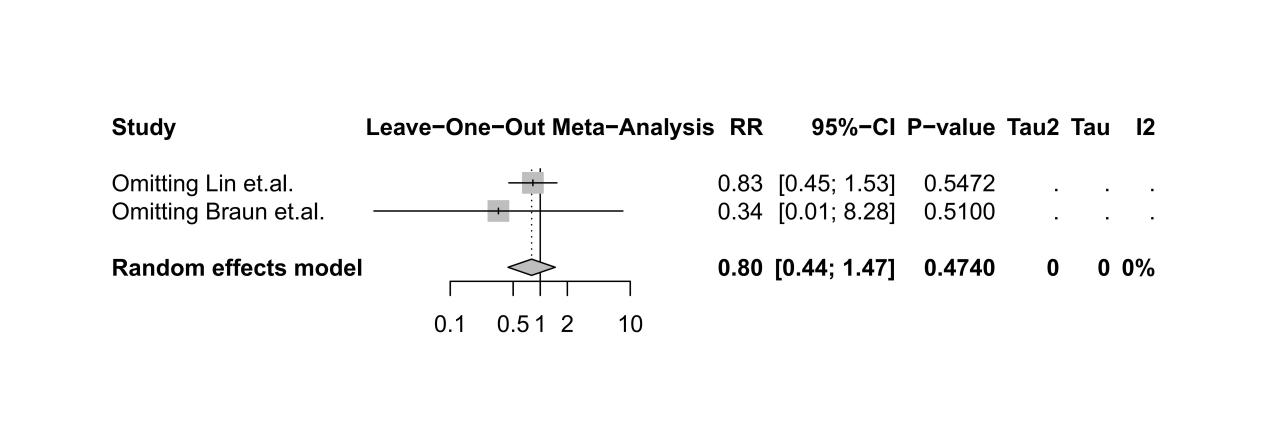
**

**D. Upper abdominal pain

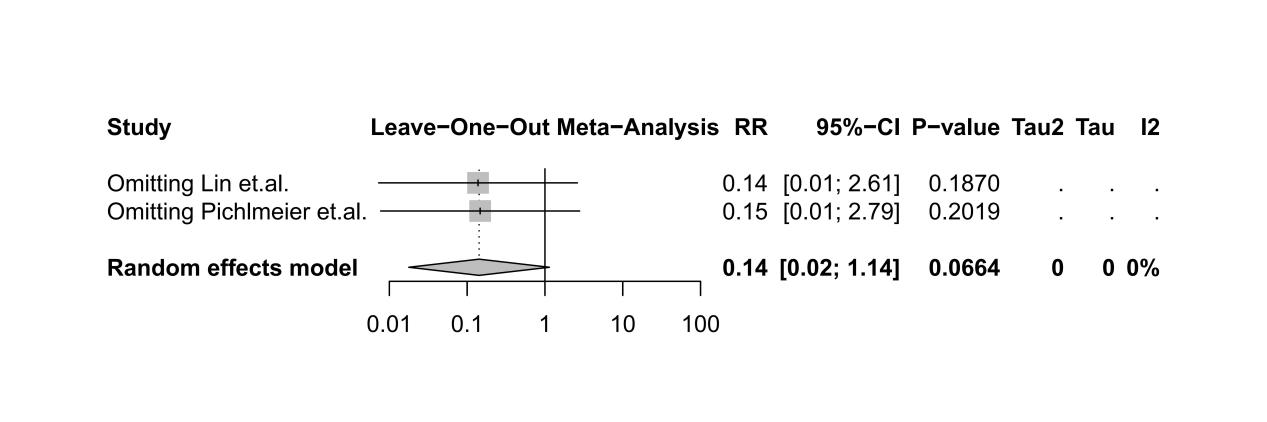
**

**E. Diarrhea

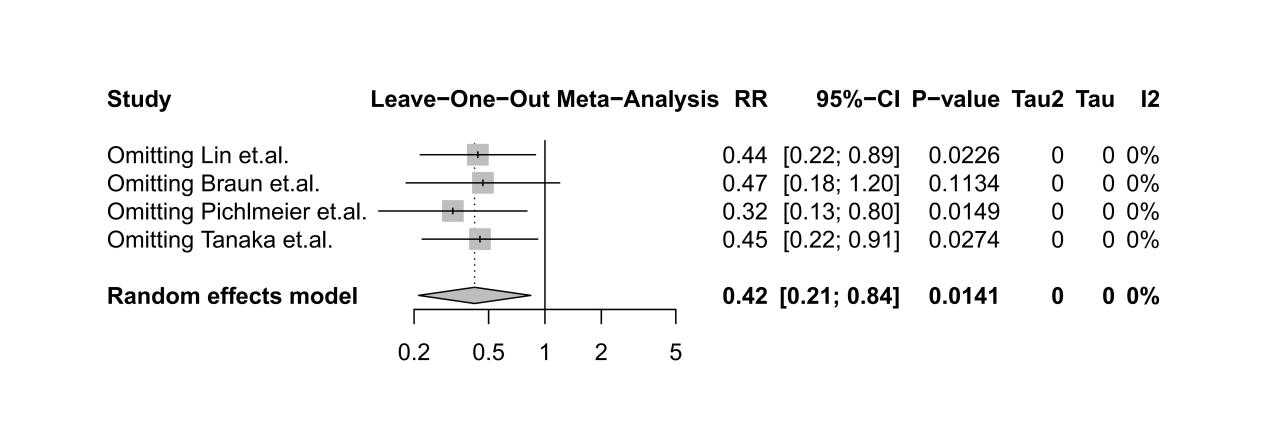
**

**F. Dyspepsia

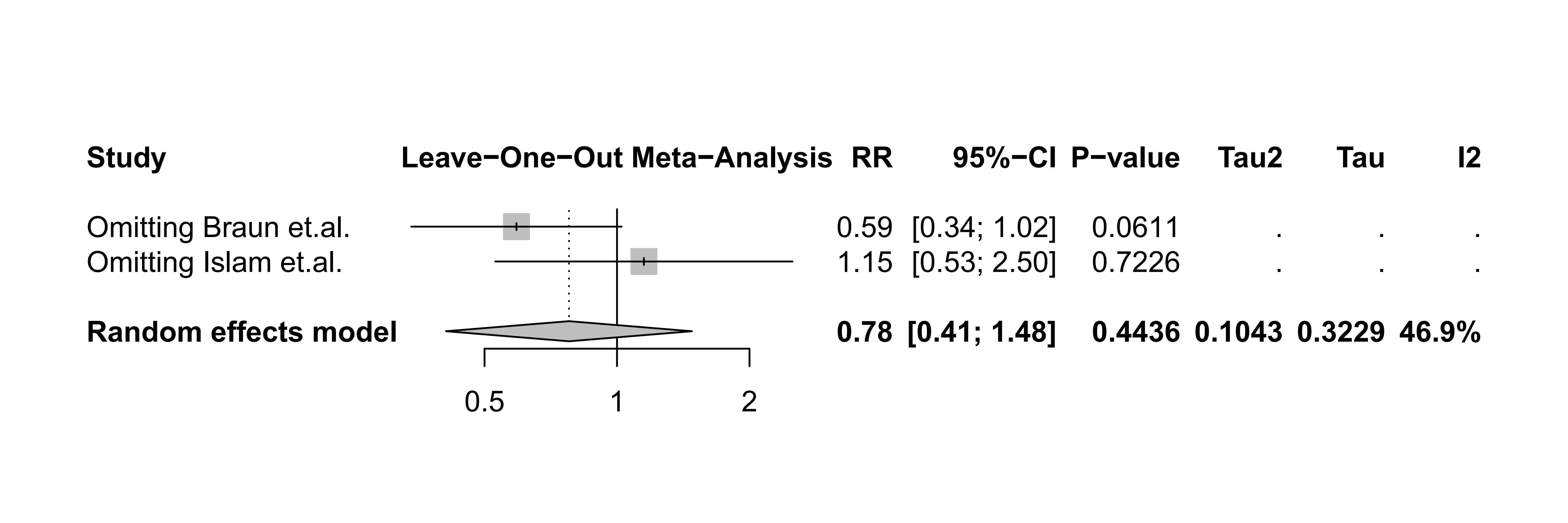
**

**G. Gastrointestinal (GI) adverse events

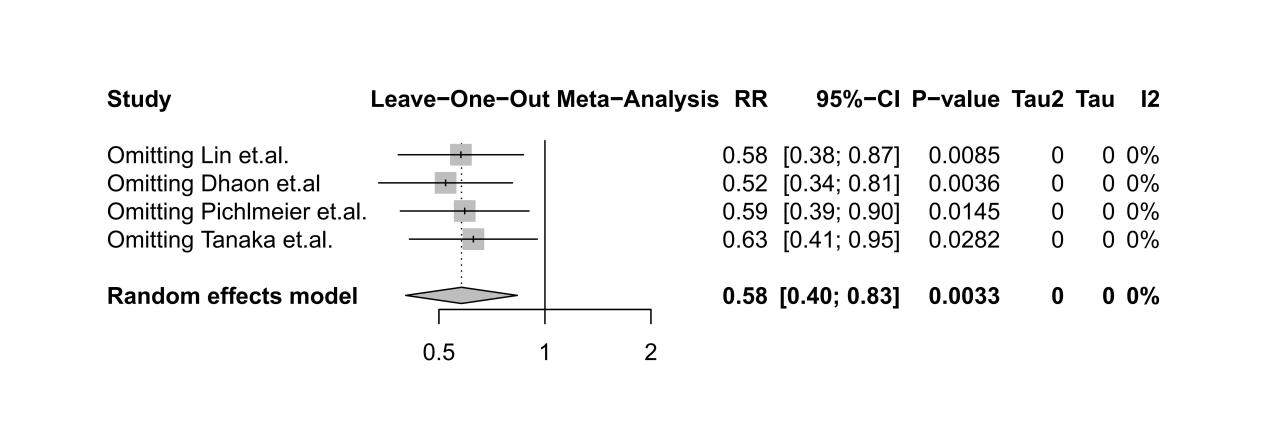
**

**H. Musculoskeletal and connective tissue disorders

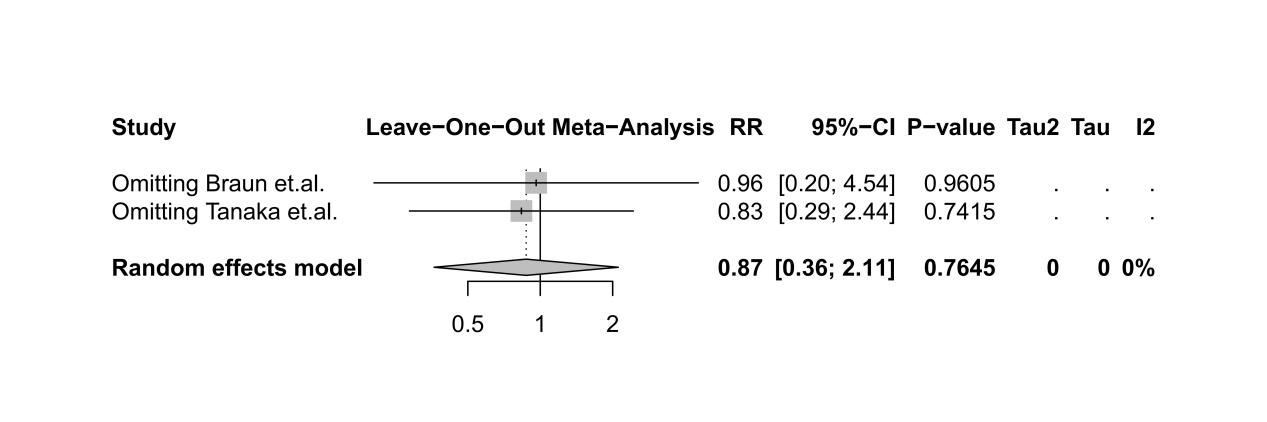
**

**I. Nausea

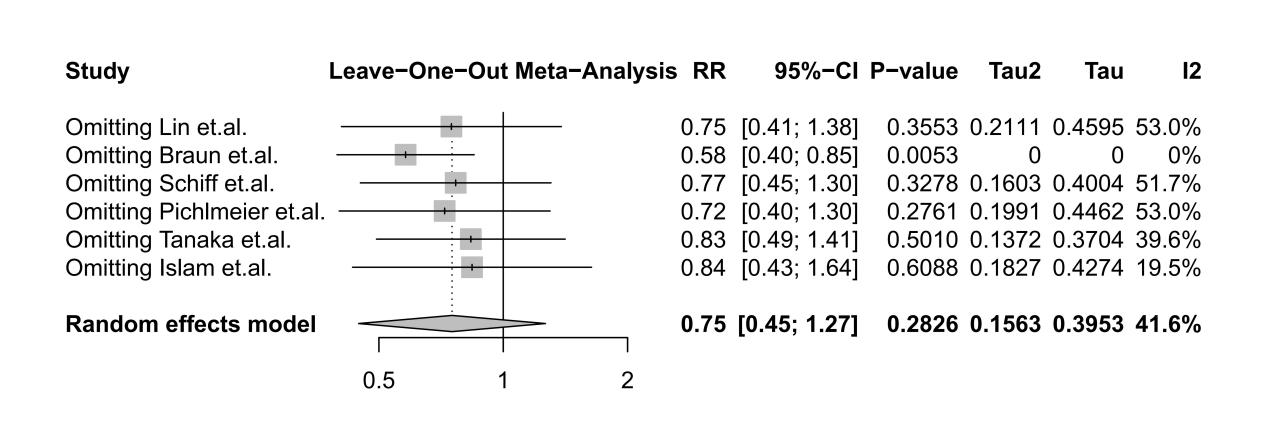
**

**J. Stomatitis**
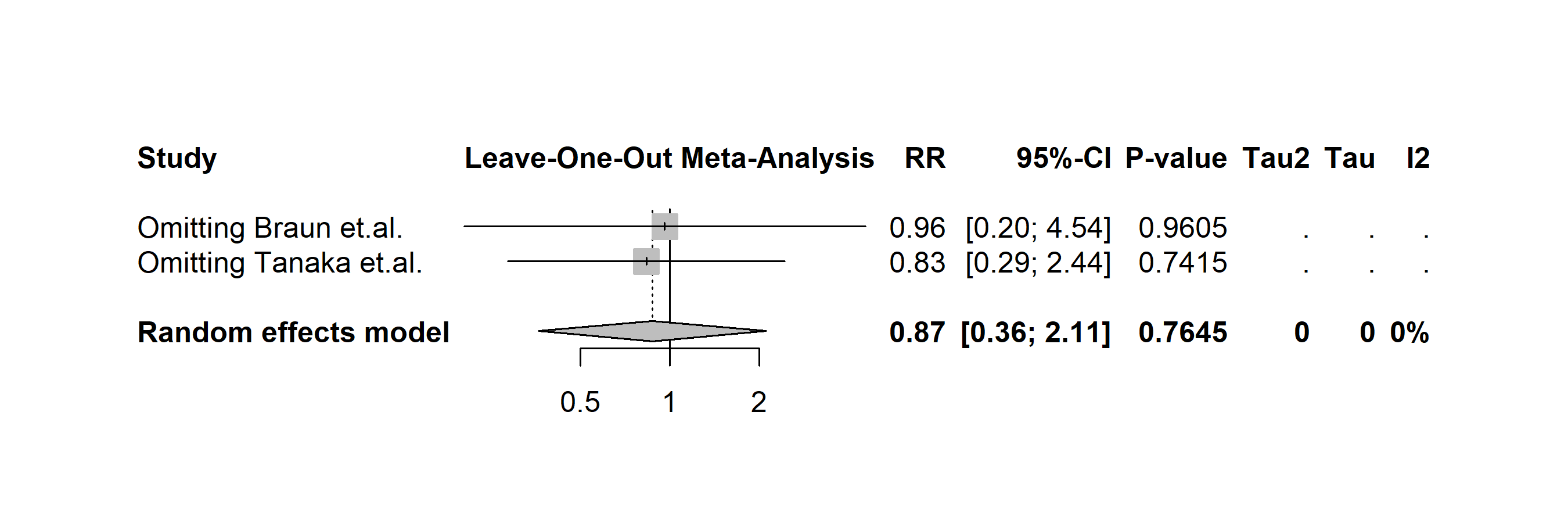


**K. Vomiting

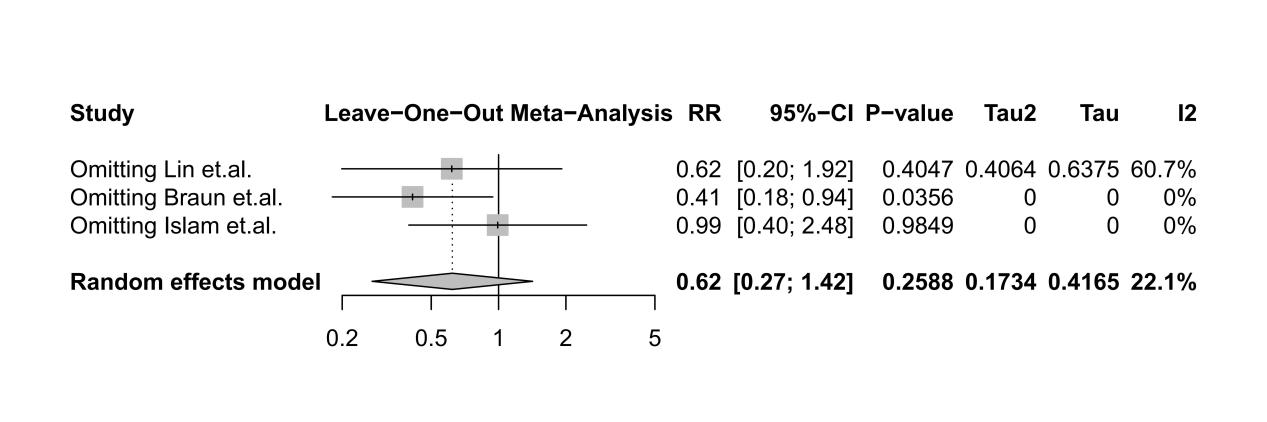
**

Figure S7. Leave-one-out sensitivity analyses for safety outcomes: (A) any adverse event (AE); (B) treatment-emergent adverse events (TEAEs); (C) abdominal pain; (D) upper abdominal pain; (E) diarrhea; (F) dyspepsia; (G) gastrointestinal (GI) adverse events; (H) musculoskeletal and connective tissue disorders; (I) nausea; (J) stomatitis; (K) vomiting.
